# The cascade of care in managing hypertension in the Arab world: a systematic assessment of the evidence on awareness, treatment and control

**DOI:** 10.1186/s12889-020-08678-6

**Published:** 2020-06-03

**Authors:** Christelle Akl, Chaza Akik, Hala Ghattas, Carla Makhlouf Obermeyer

**Affiliations:** Center for Research on Population and Health, Faculty of Health Sciences, American University of Beirut, P.O. Box: 11-0236, Riad El Solh, Beirut, 1107-2020 Lebanon

**Keywords:** Systematic assessment, Hypertension, Blood pressure, Awareness, Treatment, Control, Cascade of care, Continuum of care, Gender differences, Arab world

## Abstract

**Background:**

Hypertension is a leading risk factor for mortality and morbidity globally and in the Arab world. We summarize the evidence on awareness, treatment, and control of hypertension, to assess the extent of gaps in the hypertension continuum of care. We also assess the influence of gender and other social determinants at each level of the cascade of care.

**Methods:**

We searched MEDLINE and SSCI databases for studies published between 2000 and 2017, reporting the rates of awareness, treatment or control of hypertension and/or their determinants in the Arab region. We included sources on both general populations and on clinical populations. The review process was based on the PRISMA guidelines. We present rates on the three stages of the care cascade corresponding to (1) awareness (2) treatment and (3) control of blood pressure, and estimated the losses that occur when moving from one stage to another. We also take stock of the evidence on social determinants and assess the statistical significance of gender differences in awareness, treatment and control.

**Results:**

Data from 73 articles were included. Substantial proportions of hypertensives were lost at each step of the hypertension care continuum, with more missed opportunities for care resulting from lack of awareness of hypertension and from uncontrolled blood pressure. More than 40% and 19% of all hypertensive individuals were found to be unaware and to have uncontrolled blood pressure, respectively, but among individuals diagnosed with hypertension, less than 21% were untreated. Awareness rates were higher among women than men but this advantage was not consistently translated into better blood pressure control rates among women.

**Conclusions:**

This analysis of the cascade of care indicates that barriers to proper diagnosis and adequate control are greater than barriers to delivery of treatment, and discusses potential factors that may contribute to the gaps in delivery.

## Background

The importance of hypertension as a risk factor for cardiovascular and kidney diseases, and as a cause of mortality and morbidity is well recognized [1–3], with around 19% of global deaths and 9% of global disability-adjusted life years (DALYs) attributable to high systolic blood pressure in 2017 [4].

Cardiovascular diseases account for almost one-third of deaths in the Arab region and a 2015 analysis of data on the Eastern Mediterranean region attributed half of cardiovascular deaths to high systolic blood pressure [5]. The high prevalence of hypertension in the Arab region reflects in part the high prevalence of overweight and obesity [6–8] which have rapidly increased [9], and dire predictions have been made regarding the adverse health outcomes of these two risk factors [10]. There are however few systematic assessments of the extent to which hypertension is diagnosed and managed in the Arab region.

Global statistics indicate that despite diagnosis being straightforward and treatments widely available and relatively inexpensive, there are considerable delivery gaps at the level of awareness, treatment and control of hypertension [11, 12]. An analysis of data from 90 countries showed that 47% of all hypertensive adults were aware of their condition; 37% were being treated; and only 14% had their blood pressure under control [11], underscoring the importance of delivery gaps. In research on other diseases, most prominently HIV [13], the notion of the cascade of care has been proposed to examine the extent of delivery gaps as patients move from one stage to the next in the continuum of care [14, 15]. Few studies have applied this framework to hypertension [16, 17] and none have used it in the Arab region.

In this paper, we use the cascade of care framework to summarize the evidence on hypertension awareness, treatment and control, to identify where losses occur in the continuum of care, and to investigate to what extent social determinants influence rates of awareness, treatment and control in the different countries of the region. We pay special attention to gender differences, because the region is said to be characterized by inegalitarian gender indicators, and it is important to examine to what extent such social factors translate into health inequalities.

## Methods

### Search strategy and inclusion criteria

Our review process followed PRISMA (Preferred Reporting Items for Systematic Reviews and Meta-Analyses) guidelines, in comprehensively reviewing several electronic databases, relying on two researchers to select studies and extract data, and using clear inclusion and exclusion criteria. However, in view of the heterogeneity in the available literature and the recognized difficulty of obtaining datasets from researchers in the region, we did not aim to conduct a standard systematic review and meta-analysis. In addition, we did not limit our scope to a single question, since we wanted to explore the evidence on awareness, treatment, and control rates of hypertension. The study was thus designed to present a systematic assessment and comprehensive summary of the evidence on awareness treatment and control of hypertension in the Arab world.

We searched MEDLINE and Social Sciences Citation Index (SSCI) databases for studies, published between January 2000 and January 2017, pertaining to hypertension and its management in countries of the Arab region, defined as the 22 countries of the Arab League: Algeria, Bahrain, Comoros, Djibouti, Egypt, Iraq, Jordan, Kuwait, Lebanon, Libya, Mauritania, Morocco, Palestine, Oman, Qatar, Kingdom of Saudi Arabia (KSA), Somalia, Sudan, Syria, Tunisia, United Arab Emirates (UAE), and Yemen. Thus defined, the Arab region overlaps closely but not completely with other regional groupings, including the Eastern Mediterranean region and the North Africa and Middle East region as used in the Burden of Disease studies. Various combinations of MeSH terms and key words related to hypertension, its magnitude, burden, and social determinants were used. Details are provided in Additional file 1. We searched for additional studies manually by screening the references cited in all relevant reviews and study articles.

The selection of studies was done as a two-step process. Studies were included if they reported on any of the following: prevalence, awareness, treatment, control or management of hypertension and/or their determinants and correlates among residents of Arab countries. In this analysis, we focus on the subset of studies reporting the rates of awareness, treatment or control of hypertension and/or their determinants. Two researchers conducted title-abstract screening followed by full-text screening, to harmonize results, and resolve disagreements. They also conducted quality assessment by excluding studies that did not report on sample size, or on the methods used to measure awareness and/or treatment and/or control of hypertension; or studies that presented inconsistent numbers. We included sources on both general populations and on clinical populations. Studies published in any language were eligible. Multi-country studies were included if they presented data on at least one Arab country. Editorials, systematic reviews, meta-analyses, studies conducted exclusively on children, adolescents, or on patients undergoing treatment or suffering from particular diseases, and studies conducted on Arabs residing outside the Arab region were all excluded.

### Data extraction

Citations from search results were imported into EndNote and duplicates removed. We used the open-source Open Data Kit (ODK) software (https://ona.io/) to create the data entry protocol. Data for each study were extracted by the two independent researchers and included: article identification (title, author/s, publication year, journal, country/ies of study), research design, setting, sample size, study population, gender, and age; diagnostic methods of hypertension, and information on at least one of the following variables: awareness, treatment or control and/or their social determinants.

### Definition of hypertension, awareness, treatment and control

The available studies use different definitions and different denominators to present their results, thus requiring a careful examination of definitions and a standardized way to present our results. Most studies defined hypertension by a systolic blood pressure (SBP) > =140 mmHg and/or a diastolic blood pressure (DBP) > =90 mmHg; or by self-reported treatment for hypertension with antihypertensive medication. A few studies used different cut-off points for SBP such as > = 160 mmHg, and for DBP such as > = 80 mmHg or > =95 mmHg. In most of the studies that we retrieved, awareness was defined as self-report of any prior diagnosis of hypertension and was measured by the proportion of known hypertensives among total hypertensives; treatment was defined as use of a medication for management of hypertension at the time of the study and was reported either among all hypertensives or among aware hypertensives; control was defined as SBP < 140 mmHg and DBP < 90 mmHg and was reported among all hypertensives, aware hypertensives, or treated hypertensives. The term “undiagnosed hypertension” was sometimes used to refer to participants with no history of hypertension but who were diagnosed with hypertension in the course of surveys. For this paper, we extracted all the different denominators and terms used to calculate proportions of awareness, treatment and control (See Supplementary Table S1, Additional file 2); we also compared these proportions among men and women.

### Data analysis

**Fig. 1:**
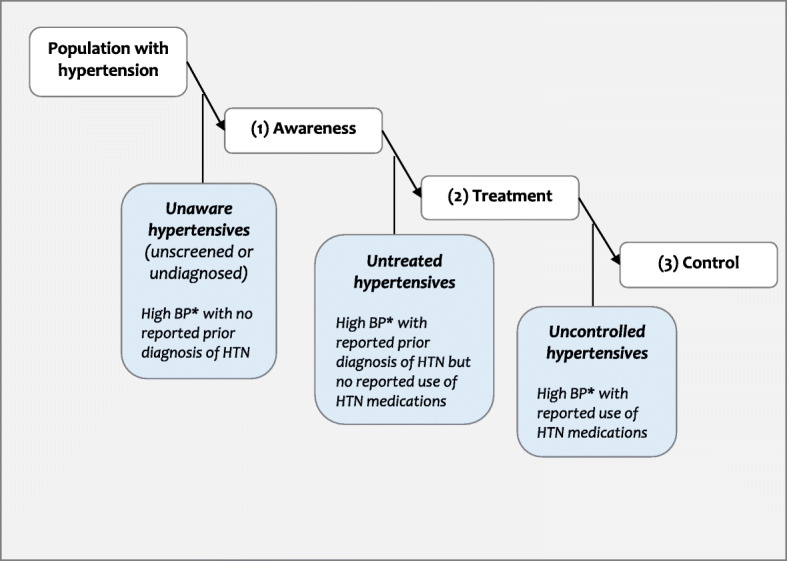
The three stages of the hypertension cascade of care showing losses that occur at different points (adapted from Berry et al, 2017 [16]). *Most studies defined hypertension by a SBP > =140 mmHg; and/or a DBP > =90 mmHg; or by a self-reported treatment with antihypertensive medication. A few studies used different cut-off points for SBP and DBP such as SBP > =140 mmHg and/or DBP > =80 mmHg; or SBP > =160 mmHg and/or DBP > =95 mmHg.

Our analysis of the cascade of care is based on cross-sectional studies, and we present results as proportions rather than survival analytical statistics which are recommended for longitudinal studies [18]. We consider three stages of the hypertension continuum of care which correspond to (1) awareness (2), treatment and (3) control, and focus on the losses that occur when moving from one stage to the other (see Fig. 1). We present rates of awareness, treatment and control as percentages and indicate whether they are based on the figures in the original publications if these were provided, or on our own calculations. For each study providing information relevant to all three stages of the hypertension continuum of care, we computed the absolute losses for each stage of the care cascade by subtracting the proportions of (1) aware hypertensives out of all hypertensives to derive the proportion unaware; (2) treated among all aware hypertensives, to derive the proportion untreated; and (3) controlled among treated hypertensives to derive the proportion uncontrolled. We also computed the relative percent losses and report the results in Supplementary Table 2 (Table S2, Additional file 3). All rates/proportions and absolute losses are presented as percentages. Similarly, we present gender ratios in awareness, treatment and control and their statistical significance, indicating whether this is based on *p*-values as reported in the original publications (crude or adjusted) or as calculated using chi-square tests (crude). Categorization of countries by income levels was based on the World Bank’s classification [19]. All statistical analyses were carried out using STATA version 13 (STATA Corporation, College Station, Texas, USA).

## Results

### State of the evidence: hypertension awareness, treatment and control in the Arab world

**Fig. 2:**
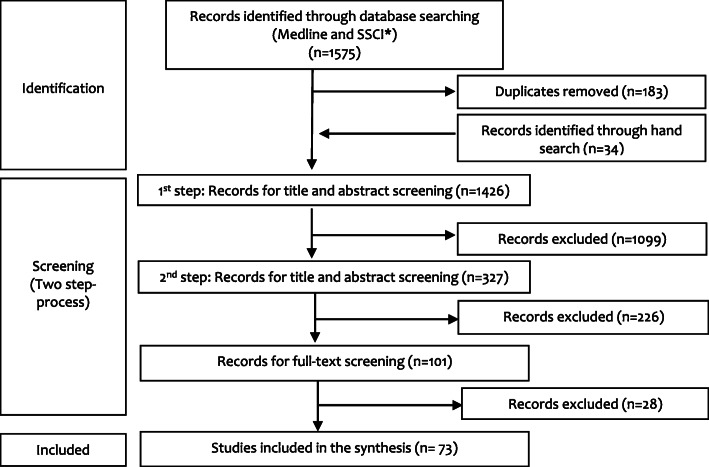
Studies’ inclusion and exclusion in the review. *SSCI: Social Sciences Citation Index (SSCI).

As shown in Fig. 2, out of 1575 retrieved publications, 73 met the inclusion criteria; these included 67 single-country and 6 multi-country studies.

Table S1 (Additional file 2) summarizes the evidence on awareness, treatment and control of hypertension by country. There were three instances where the same data source was used by two different articles [20–25]. We only kept in the analysis the three articles which provided the most information on awareness, treatment, and/or control of hypertension and/or included the different crude rates; thus a total of 70 articles contributed data. No published articles were found on Comoros, Djibouti, Iraq, Libya, Mauritania, Qatar or Somalia, and Syria lacked data on hypertension control rates. More than 3/4 of the studies were conducted on general populations and the remaining (*n* = 16/70) involved hypertensive populations. Only 17% of the articles were nationally representative studies (*n* = 12/70).

Awareness among all hypertensives ranged from 14% to 82%; the lowest rates were reported in studies conducted in Oman (18% and 24%), Morocco (22%) and a disadvantaged community in Jordan (14%) [26–29] (Table S1, Additional file 2).

Treatment rates among aware hypertensives ranged from 40% to 93%; more than 2/3 of aware hypertensives were being treated in the majority of the studies with lower rates reported in Morocco (40%), and in a disadvantaged community in Lebanon (58%) [28, 30].

Control rates among treated subjects ranged very widely, from 12% to 67%: the lowest rates were reported in studies conducted in Morocco (12%) [28], in Tunisia (13%) [31] and among older-adults in Bahrain (15%) [32] whereas the highest control rates were reported in studies conducted in Kuwait (67%) [33], Algeria (57%) [34] and Lebanon (54%) [35].

### The Cascade of hypertension care

Less than 1/3 (22/70) of articles provided information relevant to the cascade of care by (1) simultaneously including awareness, treatment and control rates of hypertension; or (2) by including enough information for the authors to calculate the corresponding values. Table 1 presents the results, using all hypertensive subjects as the denominator to facilitate comparisons.

**Fig. 3:**
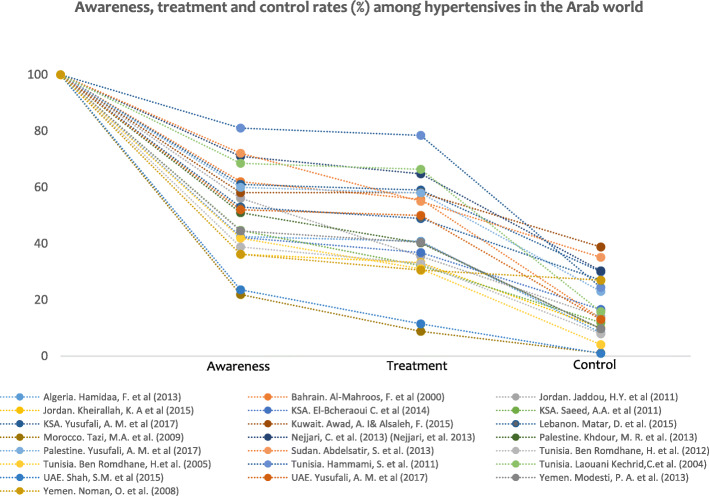
The cascade of care: Studies of awareness, treatment and control of hypertension in the Arab World, 2000–2017. Each study is represented by dots for the percent of respondents who are aware, treated, and have controlled blood pressure respectively, out of the study sample of hypertensives (as reported in the original publication). We plot a line to show how these percent decrease from one stage to the next reflecting the missed opportunities for care.

Most studies have reported measuring blood pressure several times and based on a defined protocol to ensure accuracy of the results. Results show that large proportions of hypertensives are lost at each step of the hypertension care continuum with more missed opportunities for care resulting from unawareness of hypertension and from losses between treatment and control stages (Fig. 3).

The rates presented in the following paragraphs were generated using all hypertensives as the denominator. The highest rates of awareness (81% and 69%) and treatment (78% and 66%) were observed in two studies of Tunisian older adults [36, 37]; whereas the highest rate of controlled blood pressure was observed in one study conducted in Kuwait (39%) [33]. Sudan also reported one of the highest rates of awareness (72%) and control (35%) of hypertension [38]. The multicounty study conducted in 2013 in Algeria, Morocco and Tunisia, reported high rates of awareness, treatment and control [39]. One study conducted in Morocco reported the lowest rates of all three variables: awareness (21.9%), treatment (8.8%) and control (1.1%) [28]. Control rates tend to be lower in studies conducted in households as compared to health facilities (Table 1).

Our analysis of the cascade of care was built on cross-sectional studies. In Table 1, the percent losses computed between the different stages correspond to the absolute losses from all hypertensives when moving from one stage to the other (the relative losses are presented in Table S2, Additional file 3).

In general, more than a third of all hypertensives were unaware of their hypertensive condition and were lost from the continuum of care with losses ranging from 38 to 78.1%. There were some exceptions: two studies conducted among older adults in Tunisia [36, 37], reported the lowest percentage drops at the level of awareness of hypertension (19% and 31%) but the highest percentage drops from treatment to control stages (51% and 54%); in addition, one study conducted in Sudan reported relatively lower rates of unawareness (28%) [38].

Losses between awareness and treatment stages were considerably lower overall, with less than 21% of diagnosed hypertensive patients remaining untreated. In one study conducted in Kuwait [33] no decrease was observed, indicating that diagnosed individuals were treated. There were divergent results for some countries, where different populations were studied. For example, a national study conducted in Jordan [40] reported the highest loss (21%) between awareness and treatment, while another study among an ethnic minority in the country showed only a 2% loss [41]. In Sudan, one study reported very high awareness (70%), but also a substantial drop of 17% between awareness and treatment [38].

The proportion of patients lost between treatment and control stages was greater than 19% (ranges from 19 to 54%) in all studies except in studies conducted in Yemen [42] and in Morocco [28] Substantial drops were observed in Bahrain (33%) [43]; in the Prospective Urban Rural Epidemiology (PURE) study conducted in Palestine (35%) and UAE (37%) [25]; and in the multi-country study conducted in Algeria, Tunisia and Morocco (35%) [39].

Neither hypertension awareness, treatment and control rates nor the drops within the continuum of care appear to be associated with country level of income [19]. Data from the PURE study showed that Palestine’s rates of awareness, treatment and control were higher than those reported in UAE [25]. Awareness rates were higher in Sudan [38] as compared to high-income Arab countries such as Bahrain, KSA, Kuwait and UAE [33, 43–45]. One study conducted across all governorates of Lebanon [35] showed better rates of control blood pressure than two national studies conducted in KSA [44, 45].

**Table 1:** Studies of hypertension awareness, treatment and control in the Arab countries, 2000–2017 (percent among respondents by stage and absolute-percent losses between stages)

Study	Sample size	Age^a^	Prevalence^b^ (%)	Among Hypertensives ^c^					
				Loss 1 (%)	Aware (%)	Loss 2 (%)	Treated (%)	Loss 3 (%)	Controlled (%)
** HOUSEHOLD**									
**Algeria**									
Hamidaa,F. et al. (2013) [46]	722	> = 40	50.0	* −57.5*	42.5	*−1.7*	40.8	*−32.5*	8.3
**Bahrain**									
Al-Mahroos, F. et al. (2000) ^e^ [43]	2090	40–69	30	*−38.0*	62.0	*−6.3*	55.7	*−42.3*	13.4
**Jordan**									
Jaddou, H.Y. et al. (2011)^f^ [40]	4117	> = 25	32.3	* −43.9*	56.1	*−20.6*	35.5^g^	*−21.43*	14.07^g^
Kheirallah, K. A et al. (2015) ^h^ [41]	517^g^	> = 25	44.3	*−63.8*	36.2	*−3.0*	33.2^g^	*−23.2*	10.0^g^
**KSA**									
Saeed, A.A. et al. (2011) ^f^ [45]	4758	15–64	25.5	* −55.3*	44.7	*−12.6*	32.1^g^	*−20.2*	11.9 ^g^
El-Bcheraoui C. et al. (2014) ^f^ [44]	10,735	> = 15	15.2	* −57.8*	42.2^i^	*−5.4*	36.8^i^	*−20.2*	16.6^i^
Yusufali, A. M. et al. (2017) ^j^ [25]	1545	35–70	30.0	*−39.0*	61.0^g^	* −2.0*	59.0^gf^	*−29.0*	30.0^g^
**Morocco**									
Tazi, M.A. et al. (2009) ^f^ [28]	1802	> = 20	39.6	*−78.1*	21.9	*−13.1*	8.8	*−7.7*	1.1
**Palestine**									
Yusufali, A. M. et al. (2017) ^j^ [25]	1545	35–70	37.0	*−40.0*	60.0	* −2.0*	58.0^g^	*−35.0*	23.0^g^
**Tunisia**									
Ben Romdhane, H.et al. (2005) [31]	1837	40–69	44.3	* −58.1*	41.9	*−10.9*	31.0^g^	*−26.9*	4.1^g^
Hammami, S. et al. (2011) [36]	598	> = 65	52.0	* −19.0*	81.0	*−2.6*	78.4	*−54.0*	24.4^g^
Ben Romdhane, H. et al. (2012) ^f^ [47]	8007	35–74	30.6	* −61.2*	38.8	*−5.9*	32.9^g^	*−25.0*	7.9
**UAE**									
Yusufali, A. M. et al. (2017) ^j^ [25]	1545	35–70	52.0	*−48.0*	52.0	*−2.0*	50.0^g^	*−37.0*	13.0^g^
**Yemen**									
Modesti, P. A. et al. (2013) ^f^ [20]	10,242	15–69	12.8	* −55.5*	44.5	*−4.1*	40.4	*−30.7*	9.7^g^
** HEALTH CARE FACILITIES **									
**Kuwait**									
Awad, A. I& Alsaleh, F. (2015) ^f^ [33]	1610	20–79	20.0	*−41.9*	58.1^f^	* 0*	58.1^g^	*−19.3*	38.8^g^
**Palestine (West Bank)**									
Khdour, M. R. et al. (2013) [48]	2077	25–92	27.6	*−49.0*	51.0	*−10.8*	40.2	*−30.5*	9.7
**Sudan**									
Abdelsatir, S. et al. (2013) [38]	389	41 ± 15	39.6	*−27.9*	72.1	*−17.1*	55.0^g^	*−19.9*	35.1
**Tunisia**									
Laouani Kechrid,C.et al. (2004) [37]	600	> = 60	69.3	*−31.5*	68.5	*−2.1*	66.4	*−50.6*	15.8
**Yemen**									
Noman, O. et al. (2008) [42]	994	NA	22.3^i^	*−63.8*	36.2^i^	*−5.6*	30.6^i^	*−3.6*	27.0^i^
**Multicountry study**									
Nejjari, C. et al. (2013) [39]	28,500^j^	> = 18	45.4	*−54.8*	71.0	*−6.2*	64.8^g^	*−34.5*	30.3^g^
**Other (malls, plazas, centers)**^**l**^									
**Lebanon**									
Matar, D. et al. (2015) [35]	1697	> = 21	36.9	*−47.0*	53.0	*−4.1*	48.9	*−21.9*	27.0

*NA* Not available

^a^Number of BP measurements was not mentioned in three studies (Al-Mahrous et al., 2000; Jaddou et al., 2011; Noman et al., 2008). For the other studies, BP were recorded as the average of a) 2 BP measurements (AbdelSatir et al., 2013; Awad & Alsaleh, 2015; Ben Romdhane et al., 2005; Ben Romdhane et al., 2012; Hammami et al., 2011; Khdour et al., 2013; Kheirallah et al., 2015; Nejjari et al., 2013; Shah et al., 2015; Yusufali et al., 2017), b) the last 2 BP measurements out of 3 (Hamidaa et al., 2013; Tazi et al., 2009), c) 3 BP measurements (El-Bcheraoui et al., 2014; Laouani Kechrid et al., 2004; Saeed et al., 2011), d) 2 or 3BP (Matar et al., 2015), e) 4 BP out of 6 (Modesti et al., 2013)

^b^Age is reported in years either as age group or as mean age ± standard deviation; one study did not report the sample population’s age (Noman et al., 2008)

^c^Prevalence of hypertension was identified when participants reported (1) being on current anti-hypertensive drugs and/or (2) having blood pressure measures of SBP > =160 mmHg and/or DBP > =95 mmHg for Al-Mahroos et al. (2000); SBP > =140 mmHg and/or DBP > =80 mmHg for Kheirallah et al. (2015); and SBP > =140 mmHg and/or DBP > =90 mmHg for all the remaining studies. Awareness, treatment and control were defined as the authors reported them in the original publications

^d^Proportion of patients lost when moving from one stage to another (absolute difference), calculated as follows: Loss 1 (%) = % aware - all hypertensives (i.e. 100%); Loss 2 (%) = % treated - % aware; and Loss 3 (%) = % controlled - % treated

^e^Invitations were sent to individuals at households inviting them to participate in a screening survey at health centers

^f^Nationally representative studies

^g^Estimates, based on our calculations

^h^Sample consisted of Ghawarna (an African-Descendant Ethnic Minority) living in Jordan

^i^Awareness, treatment and contol rates are weighted in the original publications (El-Bcheraoui et al., 2014; Noman et al., 2008)

^j^Crude prevalence of awareness, treatment and control retrieved from the Supplementary Table S1 (Yusufali et al., 2017)

^k^Total sample size was 28,500: Algeria (*n* = 11,905); Morocco (*n* = 10,714) & Tunisia (*n* = 5881)

^l^A study conducted by Shah et al. (2015) at a government VISA screening center reported simultaneously rates of awareness, treatment and control of hypertension. However, as the sample consists of South Asian male immigrants solely and not general populations, we did not report the results in this table

### Correlates of hypertension

In the following sections, we focus on the potentials barriers which hamper the delivery of services, and summarize the evidence on the extent to which gender and other social determinants play a role in the hypertension care cascade.

#### *Gender differences in awareness, treatment and control of* hypertension *in the Arab World*

Table 2 presents male/female (M/F) ratios for awareness, treatment and control, calculated based on the denominators used in the original publications (hypertensives, aware hypertensives or treated hypertensives). In total 27 studies reported on awareness, and/or treatment, and/or control of hypertension by gender, and yielded 20 data points for awareness, 11 for treatment and 20 for control of blood pressure.

In most studies (18/20) women are found to have greater awareness of their condition than men; this is the case in studies conducted in Jordan [40, 41, 49, 50], KSA [44, 45, 51, 52], Lebanon [35], Morocco [28], Oman [26, 27, 53], Palestine [48], Tunisia [31, 47], and Yemen [20, 54], with the difference reaching statistical significance in 11/18 studies. Only two studies, one conducted in Egypt [55] and another in KSA [56], show the reverse pattern, with a statistically significant difference.

Higher treatment rates were also observed among all hypertensive women compared to men; differences were statistically significant in 3/6 studies conducted in Algeria [46, 57] and Palestine [48]. Among the subset of hypertensives who are aware of their condition, one study in Jordan [40] reported significantly higher rates of treatment among males, while the opposite pattern was observed in Morocco [28].

Gender differences in the subsets of treated patients whose blood pressure is controlled varied across countries, and no clear patterns could be discerned; controlled blood pressure was higher among women in studies in Algeria [46, 57], Jordan [41], KSA [45], Lebanon [35], Palestine [48], and Yemen [20], but higher among men in studies conducted in Jordan [40], in Morocco [28], and three studies in Tunisia [31, 47, 58].

Rates of awareness and/or treatment and/or control were significantly higher among women in 22 cases (M/F ratio < 1); and among men in only 4 cases (M/F ratio > 1). These numbers should however be interpreted with caution, since they come from studies that reported on crude bivariate analyses, and others that reported on multivariate analyses.

**Table 2:** Gender differences in prevalence of awareness, treatment and control of hypertension in Arab countries, 2000–2017 (percent among respondents by sex, and male/female ratios)^a^

Country	Study^b^	Age^c^	Male (%)	Female (%)	Ratio M/F	Significance
**AWARENESS OF HYPERTENSION**						
**Awareness among hypertensive subjects (%)**						
**Egypt**	Mohamed, M. R. et al. (2000) [55]	> = 18	62.9	49.5	**1.27**	*P* = 0.002^e^
**Jordan**	Jaddou, H. Y. et al. (2000) [49]	> = 25	79.4	83.6	**0.95**	NS
	Jaddou, H. Y. et al. (2003) [50]	> = 25	50.0	55.6	**0.90**	*p* = 0.02
	Jaddou, H. Y. et al. (2011) [40]	> = 25	54.7	56.7	**0.96**	NS
	Kheirallah, K.A.et al (2015) [41]	> = 25	34.1	37.6	**0.91**	NS
**KSA**	Kalantan, K. A. et al. (2001) [56]	35–85	28.5	19.0	**1.50**	*P* = 0.049
	Saeed, A.A. et al. (2011) ^d^ [45]	15–64	37.3	53.3	**0.70**	*P* < 0.001
	Amin, T. T. et al. (2014) [52]	24–63	67.5	85.7	**0.79**	*P* = 0.025^e^
	El-Bcheraoui, C. et al. (2014)^f, d^ [44]	> = 15	38.8^f^	47.1^f^	**0.82**	Undiagnosed OR = 1.85[1.49–2.27]^g^, *p* < 0.05
	Mirza, A.A. et al. (2016) [51]	> = 30	49.7	62.8	**0.79**	NS
**Lebanon**	Matar, D. et al. (2015) [35]	> = 21	50.4	57.9	**0.87**	OR = 0.6 [0.4–0.9]^g^, *p* < 0.05
**Morocco**	Tazi, M.A.et al (2009) ^d^ [28]	> = 20	13.5	27.3	**0.49**	*P* < 0.001^e^
**Oman**	Al-Riyami, A. et al. (2003) ^d^ [59]	> = 20	NA	NA	**NA**	Significantly higher among females than males
	Barakat, H. et al. (2008) [53]	> = 20	14.9	21.4	**0.70**	NS^e^
	Abd El-Aty, MA. et al. (2015) ^f, d^ [27]	> = 18	13.7^f^	39.1^f^	**0.35**	Unawareness OR = 2.5 [1.7–3.3]^g^, *p* < 0.001
**Palestine**	Khdour, M. R. et al. (2013) [48]	> = 25	39.8	53.8	**0.74**	*p* < 0.01^e^
**Tunisia**	Ben Romdhane, H. et al. (2005) [31]	40–69	30.2	48.3	**0.63**	OR = 0.4 [0.3–0.5]^g^, *p* < 0.001
	Ben Romdhane, H. et al. (2012) ^d^ [47]	35–74	28.8	44.8	**0.64**	OR = 0.5 [0.4–0.6]^g^, *p* < 0.001
**Yemen**	Gunaid, A.A. et al. (2008) [54]	> = 35	25.0	38.0	**0.66**	NS^e^
	Modesti, P. A. et al. (2013) ^d^ [20]	15–69	40.7	47.6	**0.86**	NS^g^
**TREATMENT OF HYPERTENSION**						
**Treatment among hypertensive subjects (%)**						
**Algeria**	Temmar, M. et al. (2007) [57]	40–99	13.7	43.1	**0.32**	*p* < 0.0001^e^
	Hamida,F.et al. (2013) [46]	> = 40	32.0	45.0	**0.71**	*P* = 0.032^e^
**KSA**	El-Bcheraoui, C. et al. (2014) ^f, d^ [44]	> = 15	33.0^f^	42.2^f^	**0.78**	NA
**Lebanon**	Matar, D. et al. (2015) [35]	> = 20	46.1	54.0	**0.85**	NS
**Palestine**	Khdour, M. R. et al. (2013) [48]	> = 25	36.1	43.3	**0.83**	*p* = 0.02
**Yemen**	Modesti, P. A. et al. (2013) ^d^ [20]	15–69	36.5	43.6	**0.84**	NS^g^
**Treatment among aware hypertensive subjects (%)**						
**Jordan**	Jaddou, H. Y. et al. (2011) [40]	> = 25	67.0	61.9	**1.08**	*P* = 0.01
**KSA**	Saeed, A.A. et al. (2011) ^d^ [45]	15–64	74.2	69.2	**1.07**	NS
**Morocco**	Tazi, M.A. et al. (2009) ^d^ [28]	> = 20	26.3	44.9	**0.59**	*P* = 0.042^e^
**Tunisia**	Ben Romdhane, H et al. (2005) [31]	40–69	72.5	74.6	**0.97**	NS
	Ben Romdhane, H. et al. (2012)^d^ [47]	35–74	85.3	84.6	**1.01**	NS
**CONTROL OF HYPERTENSION**						
**Control among hypertensive subjects (%)**						
**KSA**	El-Bcheraoui, C. et al. (2014) ^f, d^ [44]	> = 15	13.9^f^	20.5^f^	**0.68**	NA
**Control among aware hypertensive subjects (%)**						
**KSA**	Siddiqui, S. et al. (2001) [60]	> = 18	37.7	23.8	**1.58**	NS^e^
	Mirza, A.A. et al. (2016) [51]	> = 30	37.2	39.5	**0.94**	NS
**Jordan**	Jaddou, H.Y. et al. (2000) [49]	> = 25	25.9	34.8	**0.74**	NS
	Khader, A.et al. (2014) [61]	> = 0/18	80.0	85.0	**0.94**	OR = 1.4[1.2–1.4], p < 0.001
**Oman**	Abd El-Aty, MA. et al. (2015) ^f, d^ [27]	> = 18	26.9^f^	38.3^f^	**0.70**	Uncontrolled OR = 3.3[1.4–5.0]^g^, p < 0.001
**Sudan**	Babiker, F. A. et al. (2013) [62]	> = 20	39.0	85.0	**0.46**	p < 0.001
**Algeria, Morocco Tunisia**	Nejjari, C.et al. (2013) [39]	> = 18	34.0	36.6	**0.93**	*p* = 0.01
**Control among treated hypertensive subjects (%)**						
**Algeria**	Temmar, M. et al. (2007) [57]	40–99	13.9	28.0	**0.50**	NS^e^
	Hamida,F. et al. (2013) [46]	> = 40	17.1	21.3	**0.80**	NS^e^
**Jordan**	Jaddou, H. Y. et al. (2011) [40]	> = 25	42.9	38.2	**1.12**	NS
	Kheirallah, K.A. et al. (2015) [41]	> = 25	23.3	24.5	**0.95**	NS
**KSA**	Saeed, A.A. et al. (2011) ^d^ [45]	15–64	32.0	41.1	**0.77**	NS
**Lebanon**	Matar, D.et al. (2015) [35]	> = 21	48.9	62.3	**0.78**	*p* = 0.021
**Morocco**	Tazi, M.A.et al (2009) ^d^ [28]	> = 20	20.0	11.3	**1.77**	NS^e^
**Palestine**	Khdour, M. R. et al. (2013) [48]	> = 25	29.3	35.9	**0.82**	*p* = 0.01
**Tunisia**	Ben Romdhane, H. et al. (2005) [31]	40–69	16.8	12.0	**1.40**	NS
	Masmoudi, J. et al. (2010) [58]	61.8 ± 11.9	46.3	27.1	**1.71**	*p* = 0.047
	Ben Romdhane, H. et al. (2012) ^d^ [47]	35–74	27.5	22.8	**1.21**	NS
**Yemen**	Modesti, P. A. et al. (2013) ^d^ [20]	15–69	17.2	28.8	**0.60**	OR = 0.5 [0.3–0.9]^g^, *p* < 0.05

*F* Female, *M* Male, *NA* Not available, *NS* Not significant, *OR* Odds ratio

^a^Four studies were based on hypertensive populations (Khader et al., 2014, Siddiqui et al. 2001, Babiker et al., 2013 and Masmoudi et al., 2010) while the remaining studies were based on general populations

^b^Number of BP measurements was not mentioned in 5 studies (Barakat et al., 2008; Jaddou et al., 2000; Jaddou et al., 2003; Jaddou et al., 2011; Masmoudi et al., 2010;). For the other studies, BP were recorded as the average of a) 2 BP measurements (Al Riyami et al., 2003; Amin et al., 2014; Ben Romdhane et al., 2005; Ben Romdhane et al., 2012; Gunaid et al., 2008; Kalantan et al., 2001; Kheirallah et al., 2015; Khdour et al., 2013; Mirza et al., 2016; Mohamad et al., 2000; Nejjari et al., 2013;), b) the last 2 BP measurements out of 3 (Hamidaa et al., 2013; Tazi et al., 2009; Temmar et al., 2007), c) 3 BP measurements (Abel el Aty et al., 2015; Babiker et al., 2013; El-Bcheraoui et al., 2014; Saeed et al., 2011), d) 2 or 3BP (Matar et al., 2015), e) 4 BP out of 6 (Modesti et al., 2013), f) on several separate occasions (Khader et al., 2014), and g) last 3 BP readings at least 3 months apart (Siddiqui et al., 2001)

^c^Age is reported in years either as age group or as mean age ± standard deviation

^d^Nationally representative studies

^e^Indicates that significance was calculated based on reviewer’s calculations using chi-square test for aggregated data

^f^Awareness/unawareness, controlled/uncontrolled blood pressure rates are weighted in the original publications (Abd El-Aty et al., 2015; El-Bcheraoui et al., 2014)

^g^Results of multivariate regression analyses were reported. The odd ratios for awareness/unawareness, undiagnosed, treatment and controlled/uncontrolled blood pressure are all presented with “female” being the reference variable. In Abd El-Aty et al., 2015 and El-Bcheraoui et al., 2014 odd ratios for undiagnosed hypertension, unawareness and uncontrolled blood pressure are reported

#### Other correlates of awareness, treatment and control of hypertension

Around 60% of the retrieved articles (44/70) examined some correlates of awareness and/or treatment and/or control of hypertension, but only 13/44 of these analyses adjusted for confounders; the main results of adjusted analyses are summarized below.

It is difficult to discern consistent patterns in the reported associations between social factors and hypertension awareness, treatment and control. Awareness was positively associated with: increased age [26, 27, 31, 35, 40, 47, 63]; urban residence [20, 26, 47]; utilization of health facilities [27]; family history of hypertension [63]; and other co-morbidities [26] including diabetes [35, 40, 51, 63], dyslipidemia [35], obesity [26], and heart diseases [51].

The three articles that examined correlates of treatment, found that it was positively associated with higher age [20, 35, 40].

Inconsistent results were reported for controlled blood pressure. It was not associated with other health conditions such as dyslipidemia, kidney diseases, atrial fibrillation, myocardial infarction, elevated BMI or heart diseases [40, 51, 58, 64] but some studies showed a negative association with diabetes [58, 64]and depression, anxiety and psychiatric disorders [58]. Not surprisingly, better control of blood pressure was positively associated with high adherence to anti-hypertensive treatments [58, 65] and frequent visit to health facilities [27]. Results regarding the relationship between controlled blood pressure and dietary intake indicate a negative association with indicators of poor diet, including low intake of fruits and vegetables [58], consumption of fatty and salty foods, pickles, coffee and alcohol [58, 65]. In one study, a significant association between following a diet and controlled blood pressure was found only in the sub-group of subjects suffering from both diabetes and hypertension [64].

## Discussion

This is the first review that focuses specifically on awareness, treatment and/or control rates of hypertension and their determinants in the Arab region. Out of 70 retrieved articles, only 12/70 were based on nationally representative data. Although hypertension is a leading risk factor for mortality in Iraq, Libya, Qatar and Syria, no sources on control were found for these countries.

The differences we found across countries of the region did not seem to be associated with wealth or development. Indeed, some high-income countries such as KSA or Oman had low rates of awareness, despite free and easily accessible health services and numerous prevention and health promotion campaigns [66, 67]. Similarly, the proportion of patients’ loss between treatment and control stages were reported to be similar in studies conducted in KSA [25] and Yemen [20]. Most countries of the region need to address these barriers, and even those richer countries with other cardiovascular prevention strategies and programs in place [67–69], could perhaps strengthen cardiovascular prevention by improving screening and prevention for hypertension.

We found rates of awareness, treatment and control to be generally low, and control rates were lower in household-based surveys compared to those conducted at health facilities, reflecting both the different sampling approaches and the effect of health care utilization. Most of the gaps in the care continuum were at the awareness stage and between treatment and control.

Well-known barriers to hypertension detection include the lack of knowledge regarding the importance of blood pressure screening [70] and the missed opportunity to correctly screen patients at the level of primary care facilities, where physicians may have insufficient knowledge and training, as documented in KSA [71]. The gap between treatment and control probably reflects inconvenient services [72–74], the cost of medications [75–78], and low adherence to therapy, which a number of studies have documented [17, 79, 80]. Low adherence to therapy is a contributor to uncontrolled blood pressure particularly where patients and physicians perceive lifestyle changes and stress control as sufficient. Another key factor that contributes to inadequate treatment and hence low control rates of diagnosed individuals is that health providers may not have the training and motivation to apply hypertension guidelines as documented in a study conducted in Lebanon [64]. They may also not be convinced about the thresholds used by the different guidelines to define uncontrolled blood pressure, may not be motivated to define them as goals for their patients [34, 81], or may prefer to make individual decisions about patient management [64].

The recent 2017 American Heart Association guidelines – which define hypertension as a SBP above 130 mmHg instead of 140 mmHg, and a DBP above 80 mmHg instead of 90 mmHg [82]– are expected to worsen the existent suboptimal rates of awareness, treatment and control of hypertension.

An important finding of our systematic examination of gender differences is that in almost all settings, women were more aware of their hypertension than men, and this is consistent with reports from other regions [83–87]. This could reflect women’s more frequent interactions with health care providers around reproduction and child health [84, 88], as well as the fact, reported in some studies, that primary health care facilities may be perceived as a female domain [89], and norms around masculinity discourage men from utilizing these facilities [90].

Another result of our analysis is that gender differences are themselves variable, depending on the cascade of care: indeed, while awareness was higher among women, rates of control were not always higher, suggesting that gender operates in complex rather than unidirectional ways. For example, in Morocco and Tunisia, while women are more prone to suffer from hypertension and more likely to be aware of their condition and to attend health care facilities [89], they have unfavorable statistics regarding the control of their hypertension [31, 47, 89]. This is of particular importance since it has been suggested that the reversal in the sex ratios for hypertension prevalence may occur much earlier among women in some Arab countries [25, 91]. Several factors may contribute to lower control among women, including higher rates of obesity [92] and possible differences in prescribing behaviors of health professionals.

Evidence on the social determinants and correlates of awareness, treatment and control of hypertension accounting for confounders is scarce. Studies indicate that awareness is associated with increased age and presence of co-morbidities, both of which increase the likelihood of contact with health services, and hence detection of hypertension. In addition, more favorable indicators are reported for urban residents, reflecting greater access to health services, contacts with screening campaigns, and better-informed medical personnel.

### Limitations

This paper was designed as a systematic assessment of the evidence available on the Arab world regarding awareness, treatment and control of hypertension. We did not conduct a standard systematic review and meta-analysis but our work was systematic and was based on most of the PRISMA requirements. In addition to the recognized difficulty of obtaining datasets in the Arab world, we found large discrepancies and substantial heterogeneity across the retrieved studies in regard to sample sizes, age groups, settings, and number of blood pressure measurements, which further limited our ability to pool datasets. Also, comparisons were difficult as authors were using different denominators and computation methods to estimate awareness, treatment and control rates of hypertension; thus, the evidence is patchy and pooling estimates may have yielded misleading results. Despite these limitations, our review identifies important delivery gaps in the continuum of care across all Arab countries, as well as gender differences indicating barriers for both women and men.

## Conclusions

Although hypertension is the top risk factor of premature death in the Arab World, suboptimal rates of awareness, treatment and control are observed. Considerable efforts are needed to generate consistent data, assess the magnitude of loss in the cascade of care across the different Arab countries, and identify ways to overcome existing barriers.

Our review showed substantial losses across the continuum of care particularly at the level of awareness as well as between the treatment and control levels. The similarity in rates of awareness, treatment and control of blood pressure among all hypertensives across high and low-middle income countries underscores the need to tackle delivery gaps across the region, and suggest that the main barriers are not limited to economic and resources factors. Interestingly, despite gender inequality indicators in the region, this disadvantage did not translate into greater delivery gaps.

Our paper calls for further investigations of the reasons for losses along the continuum of care, including a closer look at social determinants and gender differences, and a better understanding of the cultural context. Multipronged interventions such as screening campaigns, better information of patients and training of health providers, as well as support for treatment and, patient engagement have the potential to reduce losses across the continuum of care.

## Supplementary information

**Additional file 1.** Search Strategy. Search strategy for publications pertaining to hypertension and its management in countries of the Arab region, between January 2000 and January 2017.

**Additional file 2.** Supplementary Table 1 (Table S1). Summary of studies reporting on awareness, treatment and/or control of hypertension among general and clinical (i.e. hypertensive) populations in the Arab World, 2000–2017. List of studies included in the review and presenting evidence on hypertension awareness, treatment and control.

**Additional file 3.** Supplementary Table 2 (Table S2). Studies of hypertension awareness, treatment and control in the Arab countries, 2000–2017 (percent among respondents by stage and relative percent losses between stages). Studies of hypertension awareness, treatment and control in the Arab countries, 2000–2017 (percent among respondents by stage and relative percent losses between stages).

## Data Availability

The datasets used and/or analyzed during the current study are available from the corresponding author on reasonable request.

## Abbreviations

DALYs: Disability-adjusted life years
DBP: Diastolic blood pressure
F: Female
HIV: Human Immunodeficiency
KSA: Kingdom of Saudi Arabia
M: Male
mmHg: millimeter of mercury
NA: Not available
NS: Not significant
OR: Odds ratio
PRISMA: Preferred Reporting Items for Systematic Reviews and Meta-Analyses
PURE: Prospective Urban Rural Epidemiology
SBP: Systolic blood pressure
SSCI: Social Sciences Citation Index
UAE: United Arab Emirates

## References

[1] WHO (2013). Why hypertension is a major public health issue.

[2] Lim SS, Vos T, Flaxman AD, Danaei G, Shibuya K, Adair-Rohani H (2012). A comparative risk assessment of burden of disease and injury attributable to 67 risk factors and risk factor clusters in 21 regions, 1990–2010: a systematic analysis for the global burden of disease study 2010. Lancet (London, England).

[3] Danaei G, Lu Y, Singh G, Stevens G, Cowan M, Farzadfar F (2014). Cardiovascular disease, chronic kidney disease, and diabetes mortality burden of cardiometabolic risk factors from 1980 to 2010: a comparative risk assessment. Lancet Diabetes Endocrinol.

[4] IHME. GBD Compare Data Visualization Seattle, WA: IHME, University of Washington: Institute for Health Metrics and Evaluation; 2018 [updated (Accessed on2 February 2018). Available from: http://ghdx.healthdata.org/gbd-results-tool?params=gbd-api-2017-permalink/a1540deb2eb9b5823cf34f619075f7a6.

[5] Tehrani-Banihashemi A, Moradi-Lakeh M, Elbcheraoui C, Charara R, Khalil I, Afshin A (2018). Burden of cardiovascular diseases in the eastern Mediterranean region, 1990–2015: findings from the global burden of disease 2015 study. Int J Public Health.

[6] Chiang BN, Perlman LV, Epstein FH (1969). Overweight and hypertension: a review. Circulation..

[7] Doll S, Paccaud F, Bovet PA, Burnier M, Wietlisbach V (2002). Body mass index, abdominal adiposity and blood pressure: consistency of their association across developing and developed countries. Int J Obes.

[8] Wilson PW, D'agostino RB, Sullivan L, Parise H, Kannel WB (2002). Overweight and obesity as determinants of cardiovascular risk: the Framingham experience. Arch Intern Med.

[9] Abarca-Gómez L, Abdeen ZA, Hamid ZA, Abu-Rmeileh NM, Acosta-Cazares B, Acuin C (2017). Worldwide trends in body-mass index, underweight, overweight, and obesity from 1975 to 2016: a pooled analysis of 2416 population-based measurement studies in 128· 9 million children, adolescents, and adults. Lancet.

[10] Feigin VL, Roth GA, Naghavi M, Parmar P, Krishnamurthi R, Chugh S (2016). Global burden of stroke and risk factors in 188 countries, during 1990–2013: a systematic analysis for the global burden of disease study 2013. Lancet Neurol.

[11] Mills KT, Bundy JD, Kelly TN, Reed JE, Kearney PM, Reynolds K (2016). Global disparities of hypertension prevalence and control: a systematic analysis of population-based studies from 90 countries. Circulation..

[12] Ikeda N, Sapienza D, Guerrero R, Aekplakorn W, Naghavi M, Mokdad AH (2014). Control of hypertension with medication: a comparative analysis of national surveys in 20 countries. Bull World Health Organ.

[13] Fox MP, Rosen S (2017). A new cascade of HIV care for the era of “treat all”. PLoS Med.

[14] Perlman DC, Jordan AE, Nash D (2017). Conceptualizing care continua: lessons from HIV, hepatitis C virus, tuberculosis and implications for the development of improved care and prevention continua. Front Public Health.

[15] Gardner EM, Young B (2014). The HIV care cascade through time. Lancet Infect Dis.

[16] Berry KM, Parker W-a, Mchiza ZJ, Sewpaul R, Labadarios D, Rosen S (2017). Quantifying unmet need for hypertension care in South Africa through a care cascade: evidence from the SANHANES, 2011–2012. BMJ Glob Health.

[17] Wozniak G, Khan T, Gillespie C, Sifuentes L, Hasan O, Ritchey M (2016). Hypertension control cascade: a framework to improve hypertension awareness, treatment, and control. J Clin Hypertens.

[18] Haber N, Pillay D, Porter K, Bärnighausen T (2016). Constructing the cascade of HIV care: methods for measurement. Curr Opin HIV AIDS.

[19] World-Bank. How we classify countries. [Available from: http://data.worldbank.org/about/country-classifications.

[20] Modesti PA, Bamoshmoosh M, Rapi S, Massetti L, Al-Hidabi D, Al GH (2013). Epidemiology of hypertension in Yemen: effects of urbanization and geographical area. Hypertens Res - Clin Exp.

[21] Modesti PA, Bamoshmoosh M, Rapi S, Massetti L, Bianchi S, Al-Hidabi D (2013). Relationship between hypertension, diabetes and proteinuria in rural and urban households in Yemen. J Hum Hypertens.

[22] Youssef RM, Moubarak II (2002). Patterns and determinants of treatment compliance among hypertensive patients. East Mediterr Health J.

[23] Youssef RM, Moubarak II, Kamel MI (2005). Factors affecting the quality of life of hypertensive patients. East Mediterr Health J.

[24] Chow CK, Teo KK, Rangarajan S, Islam S, Gupta R, Avezum A (2013). Prevalence, awareness, treatment, and control of hypertension in rural and urban communities in high-, middle-, and low-income countries. Jama..

[25] Yusufali AM, Khatib R, Islam S, Alhabib KF, Bahonar A, Swidan HM (2017). Prevalence, awareness, treatment and control of hypertension in four Middle East countries. J Hypertens.

[26] Al Riyami AA, Afifi M (2003). Clustering of cardiovascular risk factors among Omani adults. East Mediterr Health J.

[27] Abd El-Aty MA, Meky FA, Morsi MM, Al-Lawati JA, El Sayed MK (2015). Hypertension in the adult Omani population: predictors for unawareness and uncontrolled hypertension. J Egypt Public Health Assoc.

[28] Tazi MA, Abir-Khalil S, Lahmouz F, Arrach ML, Chaouki N (2009). Risk factors for hypertension among the adult Moroccan population. East Mediterr Health J.

[29] Shakhatreh FM, Suleiman AA, Mohammed FI, Alwan AA (2008). Hypertension among females in a highly disadvantaged community in Jordan. Health Care Women Int.

[30] Arevian M, Adra M, Kubeissi L (2004). Risk factors for coronary artery disease (CAD) in Lebanese-Armenian women. Health Care Women Int.

[31] Ben Romdhane H, Skhiri H, Bougatef S, Ennigrou S, Gharbi D, Chahed MK (2005). Hypertension prevalence, awareness, treatment and control: results from a community based survey. Tunis Med.

[32] Al Khaja KA, Sequeira RP, Damanhori AH (2004). Pharmacotherapy and blood pressure control in elderly hypertensives in a primary care setting in Bahrain. Aging-Clin Exp Res.

[33] Awad AI, Alsaleh FM (2015). 10-year risk estimation for type 2 diabetes mellitus and coronary heart disease in Kuwait: a cross-sectional population-based study. PLoS One.

[34] Ragot S, Beneteau M, Guillou-Bonnici F, Herpin D (2016). Prevalence and management of hypertensive patients in clinical practice: cross-sectional registry in five countries outside the European Union. Blood Press.

[35] Matar D, Frangieh AH, Abouassi S, Bteich F, Saleh A, Salame E (2015). Prevalence, awareness, treatment, and control of hypertension in Lebanon. J Clin Hypertens (Greenwich, Conn).

[36] Hammami S, Mehri S, Hajem S, Koubaa N, Frih MA, Kammoun S (2011). Awareness, treatment and control of hypertension among the elderly living in their home in Tunisia. BMC Cardiovasc Disord.

[37] Laouani Kechrid C, Hmouda H, Ben Naceur MH, Ghannem H, Toumi S, Ajmi F (2004). High blood presure for people aged more than 60 years in the distrct of Sousse. Tunis Med.

[38] Abdelsatir S, Al-Sofi A, Elamin S, Abu-Aisha H (2013). The potential role of nursing students in the implementation of community-based hypertension screening programs in Sudan. Arab J Nephrol Transplant.

[39] Nejjari C, Arharbi M, Chentir MT, Boujnah R, Kemmou O, Megdiche H (2013). et al. Epidemiological trial of hypertension in North Africa (ETHNA): an international multicentre study in Algeria, Morocco and Tunisia. J Hypertens.

[40] Jaddou H, Batieha A, Khader YS, Kanaan A, El-Khateeb M, Ajlouni K (2011). Hypertension prevalence, awareness, treatment and control, and associated factors: results from a national survey. Jordan. Int J Hypertens..

[41] Kheirallah KA, Liswi M, Alazab R, Bataineh Z, Alzyoud S, Alsulaiman J (2015). et al. Hypertension prevalence, awareness and control levels among Ghawarna: an African-descendant ethnic minority in the Jordan Valley. Ethn Dis.

[42] Noman O, Al-Kaddoomi SA, Del Ben M, Angelico F (2008). Impact of urbanization on the prevalence and pattern of arterial hypertension on the island of Socotra. Ann Saudi Med.

[43] Al-Mahroos F, Al-Roomi K, McKeigue PM (2000). Relation of high blood pressure to glucose intolerance, plasma lipids and educational status in an Arabian gulf population. Int J Epidemiol.

[44] El Bcheraoui C, Memish ZA, Tuffaha M, Daoud F, Robinson M, Jaber S (2014). Hypertension and its associated risk factors in the Kingdom of Saudi Arabia, 2013: a national survey. Int J Hypertens.

[45] Saeed AA, Al-Hamdan NA, Bahnassy AA, Abdalla AM, Abbas MA, Abuzaid LZ (2011). Prevalence, awareness, treatment, and control of hypertension among Saudi adult population: a National Survey. Int J Hypertens..

[46] Hamida F, Atif ML, Temmar M, Chibane A, Bezzaoucha A, Bouafia MT (2013). Prevalence of hypertension in El-Menia oasis, Algeria, and metabolic characteristics in population. Annales de Cardiologie et d Angeiologie.

[47] Ben Romdhane H, Ben Ali S, Skhiri H, Traissac P, Bougatef S, Maire B (2012). Hypertension among Tunisian adults: results of the TAHINA project. Hypertens Res Clin Exp.

[48] Khdour MR, Hallak HO, Shaeen M, Jarab AS, Al-Shahed QN (2013). Prevalence, awareness, treatment and control of hypertension in the Palestinian population. J Hum Hypertens.

[49] Jaddou HY, Bateiha AM, Ajlouni KM (2000). Prevalence, awareness and management of hypertension in a recently urbanised community, eastern Jordan. J Hum Hypertens.

[50] Jaddou HY, Bateiha AM, Al-Khateeb MS, Ajlouni KM (2003). Epidemiology and management of hypertension among Bedouins in northern Jordan. Saudi Med J.

[51] Mirza AA, Elmorsy SA (2016). Diagnosis and control of hypertension as indicators of the level of awareness among relatives of medical students in Saudi Arabia. High Blood Pressure Cardiovascr Prev.

[52] Amin TT, Al Sultan AI, Mostafa OA, Darwish AA, Al-Naboli MR (2014). Profile of non-communicable disease risk factors among employees at a Saudi university. Asian Pac J Cancer Prev.

[53] Barakat MN, Youssef RM (2008). Prevalence of dysglycemia and other cardiovascular risk factors among the rural population of Oman. Saudi Med J.

[54] Gunaid AA, Assabri AM (2008). Prevalence of type 2 diabetes and other cardiovascular risk factors in a semirural area in Yemen. East Mediterr Health J.

[55] Mohamed MR, Shafek M, El Damaty S, Seoudi S (2000). Hypertension control indicators among rural population in Egypt. J Egypt Public Health Assoc.

[56] Kalantan KA, Mohamed AG, Al-Taweel AA, Abdul Ghani HM (2001). Hypertension among attendants of primary health care centers in Al-Qassim region. Saudi Arabia. Saudi Med J..

[57] Temmar M, Labat C, Benkhedda S, Charifi M, Thomas F, Bouafia MT (2007). et al. Prevalence and determinants of hypertension in the Algerian Sahara. J Hypertens.

[58] Masmoudi J, Imene T, Ketata W, Mnif L, Maalej S, Kammoun S (2010). et al. Role of the psychosocial factors in blood pression balance; cross-sectional study including 100 ambulatory hypertensive patients. Tunis Med.

[59] Al-Riyami AA, Afifi MM (2003). Accuracy of self-reporting of diabetes mellitus and hypertension and its determinants among Omani adults. Saudi Med J.

[60] Siddiqui S, Ogbeide DO, Karim A, Al-Khalifa I (2001). Hypertension control in a community health Centre at Riyadh. Saudi Arabia. Saudi Med J..

[61] Khader A, Farajallah L, Shahin Y, Hababeh M, Abu-Zayed I, Zachariah R (2014). et al. Hypertension and treatment outcomes in Palestine refugees in United Nations relief and works agency primary health care clinics in Jordan. Tropical Med Int Health.

[62] Babiker FA, Elkhalifa LA, Moukhyer ME (2013). Awareness of hypertension and factors associated with uncontrolled hypertension in Sudanese adults. Cardiovasc J Afr.

[63] Shah SM, Loney T, Sheek-Hussein M, El Sadig M, Al Dhaheri S, El Barazi I (2015). Hypertension prevalence, awareness, treatment, and control, in male south Asian immigrants in the United Arab Emirates: a cross-sectional study. BMC Cardiovasc Disord.

[64] Mallat SG, Samra SA, Younes F, Sawaya MT (2014). Identifying predictors of blood pressure control in the Lebanese population - a national, multicentric survey -- I-PREDICT. BMC Public Health.

[65] El-Badawy AM, Al-Kharusi HM, Al-Ghanemy SA (2005). Health habits and risk factors among Omanis with hypertension. Saudi Med J.

[66] El Bcheraoui C, Tuffaha M, Daoud F, Kravitz H, AlMazroa MA, Al Saeedi M (2015). Access and barriers to healthcare in the Kingdom of Saudi Arabia, 2013: findings from a national multistage survey. BMJ Open.

[67] Khoja T, Rawaf S, Qidwai W, Rawaf D, Nanji K, Hamad A (2017). Health care in gulf cooperation council countries: a review of challenges and opportunities. Cureus.

[68] Hajat C, Harrison O, Al SZ (2012). Weqaya: a population-wide cardiovascular screening program in Abu Dhabi, United Arab Emirates. Am J Public Health.

[69] Health KoBMo (2015). National Strategy for control and prevention of non - communicable diseases in Kingdom of Bahrain 2014–2025.

[70] Khatib R, Schwalm J-D, Yusuf S, Haynes RB, McKee M, Khan M (2014). Patient and healthcare provider barriers to hypertension awareness, treatment and follow up: a systematic review and meta-analysis of qualitative and quantitative studies. PLoS One.

[71] Al-Gelban KS, Khan MY, Al-Khaldi YM, Mahfouz AA, Abdelmoneim I, Daffalla A (2011). Adherence of primary health care physicians to hypertension management guidelines in the Aseer region of Saudi Arabia. Saudi J Kidney Dis Transplant.

[72] Murimi MW, Harpel T (2010). Practicing preventive health: the underlying culture among low-income rural populations. J Rural Health.

[73] Ogedegbe G, Harrison M, Robbins L, Mancuso CA, Allegrante JP (2004). Barriers and facilitators of medication adherence in hypertensive African Americans: a qualitative study. Ethn Dis.

[74] Buzza C, Ono SS, Turvey C, Wittrock S, Noble M, Reddy G (2011). Distance is relative: unpacking a principal barrier in rural healthcare. J Gen Intern Med.

[75] El Zubier A (2000). Drug compliance among hypertensive patients in Kassala, eastern Sudan.

[76] Schafheutle EI, Hassell K, Noyce PR, MRPharmS MCWD (2002). Access to medicines: cost as an influence on the views and behaviour of patients. Health Soc Care Community.

[77] Tohme R, Jurjus A, Estephan A (2005). The prevalence of hypertension and its association with other cardiovascular disease risk factors in a representative sample of the Lebanese population. J Hum Hypertens.

[78] Al-Ramahi R (2015). Adherence to medications and associated factors: a cross-sectional study among Palestinian hypertensive patients. J Epidemiol Glob Health.

[79] Chobanian AV, Bakris GL, Black HR, Cushman WC, Green LA, Izzo JL (2003). The seventh report of the joint national committee on prevention, detection, evaluation, and treatment of high blood pressure: the JNC 7 report. Jama..

[80] Choudhry NK, Fischer MA, Avorn J, Liberman JN, Schneeweiss S, Pakes J (2011). The implications of therapeutic complexity on adherence to cardiovascular medications. Arch Intern Med.

[81] Bovet P, Chiolero A (2018). Prevalence and control of hypertension. Lancet.

[82] Whelton PK, Carey RM, Aronow WS, Casey DE, Collins KJ, Himmelfarb CD (2018). 2017 ACC/AHA/AAPA/ABC/ACPM/AGS/APhA/ASH/ASPC/NMA/PCNA guideline for the prevention, detection, evaluation, and management of high blood pressure in adults: a report of the American College of Cardiology/American Heart Association task force on clinical practice guidelines. J Am Coll Cardiol.

[83] Yang L, Yan J, Tang X, Xu X, Yu W, Wu H (2016). Prevalence, awareness, treatment, control and risk factors associated with hypertension among adults in southern China, 2013. PLoS One.

[84] Everett B, Zajacova A (2015). Gender differences in hypertension and hypertension awareness among young adults. Biodemography Soc Biol.

[85] Abdul-Razak S, Daher AM, Ramli AS, Ariffin F, Mazapuspavina MY, Ambigga KS (2016). Prevalence, awareness, treatment, control and socio demographic determinants of hypertension in Malaysian adults. BMC Public Health.

[86] Doumas M, Papademetriou V, Faselis C, Kokkinos P (2013). Gender differences in hypertension: myths and reality. Curr Hypertens Rep.

[87] Chor D, Ribeiro ALP, Carvalho MS, Duncan BB, Lotufo PA, Nobre AA (2015). Prevalence, awareness, treatment and influence of socioeconomic variables on control of high blood pressure: results of the ELSA-Brasil study. PLoS One.

[88] Zhang Y, Moran AE (2017). Trends in the prevalence, awareness, treatment, and control of hypertension among young adults in the United States, 1999 to 2014. Hypertension..

[89] Alberti H, Alberti B (2009). The influence of gender on the primary care management of diabetes in Tunisia. Pan Afr Med J.

[90] Yousaf O, Grunfeld EA, Hunter MS (2015). A systematic review of the factors associated with delays in medical and psychological help-seeking among men. Health Psychol Rev.

[91] Akl C, Akik C, Ghattas H, Obermeyer CM (2017). Gender disparities in midlife hypertension: a review of the evidence on the Arab region. Women’s Midlife Health.

[92] Kanter R, Caballero B (2012). Global gender disparities in obesity: a review. Adv Nutr.

