# Supplementary material for: The cascade of care in managing hypertension in the Arab world: a systematic assessment of the evidence on awareness, treatment and control
Source: BMC Public Health. 2020 Jun 3;20:835. doi: 10.1186/s12889-020-08678-6 (PMC7268748; doi:10.1186/s12889-020-08678-6)
Supplement: Supplementary file 1 — Additional file 1. Search Strategy. Search strategy for publications pertaining to hypertension and its management in countries of the Arab region, between January 2000 and January 2017. [file 12889_2020_8678_MOESM1_ESM.docx]

**Additional file 1**

**Medline Search strategy**

[[Hypertension/ or hypertensi*.mp. or HTN.mp. or Blood Pressure/ or (systolic adj2 pressure).mp. or (diastolic adj2 pressure).mp. or BP.mp. or (SBP or DBP).mp. or (blood adj2 pressure).mp.

NOT

gestational hypertension.mp. or exp Hypertension, Pregnancy-Induced/]

AND

Arab world/ or Arabs.mp. or Algeria/ or Algeria*.mp. or Bahrain/ or

Bahrain*.mp. or Comoros/ or Comoros.mp. or Comorian*.mp. or Djibouti/ or

Djibouti.mp. or Djiboutian*.mp. or Egypt/ or Egyptian*.mp. or Iraq/ or Iraqi*.mp.

or Jordan/ or Jordanian*.mp. or Kuwait/ or Kuwait*.mp. or Lebanon/ or

Lebanese.mp. or Libya/ or Libya*.mp. or Mauritania/ or Mauritania*.mp. or

Morocco/ or Morocco*.mp. or Moroccan*.mp. or Oman/ or Oman*.mp. or

Palestine/ or Palestin*.mp. or occupied Palestinian territor*.mp. or West Bank.mp.

or Gaza.mp. or Qatar/ or Qatar*.mp. or Saudi Arabia/ or Saudi*.mp. or Somalia/

or Somali*.mp. or Sudan/ or Sudan*.mp. or Syria/ or Syria*.mp. or Tunisia/ or

Tunisia*.mp. or United Arab Emirates.mp. or Emirati*.mp. or Yemen/ or

Yemen*.mp. or gulf cooperation council.mp. or gulf countr*.mp. or middle

East*.mp. or Middle East/ or Africa, Northern/ or North Africa*.mp. or (Middle

East and North Africa).mp.

AND

[epidemiology/ or epidemiolog*.mp or prevalence/ or prevalen*.mp or incidence/ or inciden*.mp or Vital Statistics or vital statistics.mp

OR

Burden of disease.mp or disease burden.mp or morbidity/ or morbidity.mp or

mortality/ or "cause of death"/ or mortality, premature/ or survival rate/or Quality-

Adjusted Life Years/ or quality-adjusted life years.mp or QALY.mp or disabilityadjusted

life years.mp or DALY.mp or YLD.mp or years of life lost.mp or YLL.mp or years lived with disability.mp or years lived with disease.mp or cost of illness/

OR

(social or socio* or cultur*).mp.]]

NOT

exp genetic phenomena/

Limit: 2000- current

**SSCI search strategy**

[TS=hypertensi* OR TS=HTN OR TS="blood pressure" OR TS="systolic pressure" OR TS="systolic blood pressure" OR TS="diastolic pressure" OR TS="diastolic blood pressure" OR TS="pulse pressure"

NOT

TS="gestational hypertensi*" OR TS="Pregnancy-Induced Hypertensi*" OR TS=eclampsia or TS=pre-eclampsia

AND

TS= "Arab world" OR TS="Arab countr*" OR TS=Arabs OR TS=Algeria* OR TS=Bahrain* OR TS= Comoros OR TS=Comorian* OR TS= Djibouti* OR TS=Egypt* OR TS= Iraq* OR TS=Jordan* OR TS=Kuwait* OR TS= Leban* OR TS=Libya* OR TS=Mauritania* OR TS=Morocc* OR TS=Oman* OR TS=Palestin* OR TS= "Occupied Palestinian Territor*" OR TS= Gaza OR TS="West Bank" OR TS= Qatar* OR TS=Saudi Arabia* OR TS=Somalia* OR TS=Sudan* OR TS=Syria* OR TS=Tunisia* OR TS=United Arab Emirates OR TS=Emirati* OR TS=Yemen* OR TS="Gulf cooperation council" OR TS= "Gulf countr*" OR TS="Middle East*" OR TS="North Africa*"

AND

[TS=epidemiolog* OR TS=inciden* OR TS=prevalen* OR TS="vital statistics"

OR

TS=morbidity OR TS=mortality OR TS="quality-adjusted life years" OR TS=QALY OR TS="disability-adjusted life years" OR TS=DALY OR TS="years lived in disease" OR TS="years lived in disability" OR TS=YLD OR TS="years of life lost" OR TS=YLL OR TS="cost of illness"

OR

TS=Social* OR TS=Socio* OR TS=Cultur*]
