# Supplementary material for: The cascade of care in managing hypertension in the Arab world: a systematic assessment of the evidence on awareness, treatment and control
Source: BMC Public Health. 2020 Jun 3;20:835. doi: 10.1186/s12889-020-08678-6 (PMC7268748; doi:10.1186/s12889-020-08678-6)
Supplement: Supplementary file 2 — Additional file 2. Supplementary Table 1 (Table S1). Summary of studies reporting on awareness, treatment and/or control of hypertension among general and clinical (i.e. hypertensive) populations in the Arab World, 2000–2017. List of studies included in the review and presenting evidence on hypertension awareness, treatment and control. [file 12889_2020_8678_MOESM2_ESM.docx]

**Additional file 2**

**Supplementary Table 1: Summary of studies reporting on awareness, treatment and/or control of hypertension among general and clinical (i.e. hypertensive) populations in the Arab World, 2000-2017 ^*^**

| **Country** | **First Author (year)** | **Sample size** | **Age**  *(age group or*  *mean age± SD)* |  | **Prevalence of awareness (%)**  **among hypertensives** |  | **Prevalence of treatment (%)** | |  | **Prevalence of control (%)** | | |
| --- | --- | --- | --- | --- | --- | --- | --- | --- | --- | --- | --- | --- |
|  |  |  |  |  |  |  | **Among hypertensives** | **Among aware hypertensives** |  | **Among hypertensives** | **Among aware hypertensives** | **Among treated hypertensives** |
| **PANEL A: GENERAL POPULATION** | | | | | | | | | | | | |
| **Setting: Household** | | | | | | | | | | | | |
| Algeria | Temmar, M.et al (2007) ^(1)^ | 1346 | 40-99 |  |  |  | 30.0 |  |  |  |  | 25.0 |
|  | Hamida, F. et al (2013) ^(2)^ | 722 | >=40 |  | 42.5 |  | 41.0 |  |  |  |  | 20.0 |
| Bahrain | Al-Mahroos, F.et al (2000) ^(3)^ | 2090 | 40-69 |  | 62.0 |  |  | 90.0 |  |  | 22.0 |  |
| Egypt | Mohamed, M. R.et al (2000) ^(4)^ | 1927 | >=18 |  | 55.5 |  |  |  |  |  | 24.2 |  |
| Jordan | Jaddou, H. Y.et al (2000) ^(5)^ | 545 | >=25 |  | 82.0 |  |  |  |  |  | 31.5 |  |
|  | Jaddou, H. Y et al (2003) ^(6)^ | 366 | >=25 |  | 52.5 |  |  |  |  |  | 42.9 |  |
|  | Shakhatreh, F. M. et al (2008) ^(7)^ | 462  *(only females)* | 15-49 |  | 14 .0 |  |  |  |  |  |  |  |
|  | Jaddou, H. Y. et al (2011) ^**(8)^ | 4117 | >=25 |  | 56.1 |  |  | 63.3 |  |  |  | 39.6 |
|  | Kheirallah, K. A. et al (2015) ^(9)^ | 517 | >=25 |  | 36.2 |  |  | 91.5 |  | 10.0 | 27.7 | 23.7 |
| KSA | Al-Nozha, M. M.et al (2007) ^**(10)^ | 17230 | 30-70 |  | 33.1 |  |  |  |  |  |  | 25.0 |
|  | Saeed, A. A. et al (2011)^**(11)^ | 4758 | 15-64 |  | 44.7 |  | 32.1 | 71.8 |  |  |  | 37.0 |
|  | El Bcheraoui, C. et al (2014) ^** (12)^ | 10735 | >=15 |  | 42.2 ^†^ |  | 36.8 ^†^ | 78.9 ^†^ |  | 16.6^†^ |  | 45.0 ^†^ |
|  | Mirza, A. A.et al (2016) ^(13)^ | 770 | >=30 |  | 53.9 |  |  |  |  |  | 38.1 |  |
|  | Yusufali, A. M. et al (2017) ^§(14)^ | 2041 | 35-70 |  | 6.01 ^‡^ |  | 59.0 ^‡^ |  |  | 30.0 ^‡^ |  |  |
| Morocco | Tazi, M. A.et al (2009) ^**(15)^ | 1802 | >=20 |  | 21.9 |  | 8.8 | 40.4 |  | 1.1 |  | 12.7 |
| Oman | Al-Riyami, A. A. et al (2003) ^**(16)^ | 6414 | >=20 |  | 18.4 |  |  |  |  |  |  |  |
|  | Abd El-Aty, M. A. et al (2015) ^**(17)^ | 3478 | >=18 |  | 24.5^††^ |  |  | 82.7 |  |  | 34.5 ^††^ |  |
| Syria | Maziak, W.et al (2007) ^(18)^ | 2038 | 18-65 |  | 29.0 |  |  | 72.8 |  |  |  |  |
| Tunisia | Ben Romdhane, H.et al (2005) ^(19)^ | 1837 | 40-69 |  | 41.9 |  |  | 74.1 |  |  |  | 13.2 |
|  | Hammami, S. et al (2011) ^(20)^ | 598 | >=65 |  | 81.0 |  |  | 78.4 |  |  |  | 30.7 |
|  | Ben Romdhane, H. et al (2012) ^**(21)^ | 8007 | 35-74 |  | 38.8 |  |  | 84.8 |  |  |  | 24.1 |
| Palestine | Yusufali, A. M. et la (2107) ^§(14)^ | 1545 | 35-70 |  | 60.0^‡^ |  | 58 ^‡^ |  |  | 23.0 ^‡^ |  |  |
| UAE | Yusufali, A. M. et al (2017) ^§(14)^ | 917 | 35-70 |  | 52.0 ^‡^ |  | 50 ^‡^ |  |  | 13.0^‡^ |  |  |
| Yemen | Gunaid, A. A.et al (2008) ^(22)^ | 250 | 35-64 |  | 30.8 |  |  | 70.0 |  |  |  |  |
|  | Modesti, P. A. et al (2013) ^**(23)^ | 10242 | 15-69 |  | 44.5 |  | 40.4 |  |  |  |  | 24.1 |
| **Setting : Health care facilities** | | | | | | | | | | | | |
| Algeria | Temmar, M. et al (2013) ^§ ∫(24)^ | 321 | >=74 |  |  |  | 49.0 |  |  |  |  |  |
|  | Bachir Cherif, A. et al (2016) ^(25)^ | 1721  *(only females)* | 65.3± 9.5 |  |  |  |  |  |  | 31.7 |  |  |
| Bahrain | Whitford, D. L. et al (2013) ^(26)^ | 334 | 55± 9.5 |  |  |  |  |  |  | 34.0 |  |  |
| KSA | Al-Homrany, M. A. et al (2008) ^(27)^ | 120 | NA |  |  |  |  |  |  | 63,0 |  |  |
|  | Kalantan, K. A. et al (2001)^(28)^ | 1114 | 35-85 |  | M: 28.5; F: 19.0 |  |  |  |  |  |  |  |
| Kuwait | Awad, A. I.et al (2015) ^**(29)^ | 1610 | 20-79 |  | 58.0 |  | 58.0 ^j^ |  |  | 38.8 |  | 66.8 ^j^ |
| Lebanon | Arevian, M. et al (2004) ^(30)^ | 76  *(only females)* | >=25 |  | 41 .0 |  |  | 58.8 |  |  |  |  |
| Oman | Barakat, M. N. et al (2008) ^(31)^ | 879 | >=20 |  | 19.3 |  |  |  |  |  |  |  |
| Palestine | Khdour, M. Ret al (2013) ^(32)^ | 2077 | 25-92 |  | 51.0 |  | 40.2 |  |  | 9.7 |  | 33.2 |
| Sudan | Abdelsatir, S.et al (2013) ^(33)^ | 389 | 41.± 15 |  | 72.1 |  | 55.0 | 80.2 |  |  | 48.6 |  |
| Tunisia | Laouani Kechrid, C.et al (2004) ^(34)^ | 260 | >=60 |  | 68.5 |  | 64.4 |  |  | 15.8 |  |  |
| UAE | Abdulle, A. M.et al (2006) ^(35)^ | 989 | 20-75 |  | 61.7 |  | 24.0 |  |  |  |  |  |
| Yemen | Noman, O. et al (2008) ^(36)^ | 994 | NA |  | 36.2 |  | 30.6 |  |  | 27.0 |  |  |
| Multi-countries study | Nejjari, C.et al (2013) ^§(37)^ | 28500 | >=18 |  | 71.0^†^ |  |  | 91.2^†^ |  |  | 35.7^†^ |  |
| **Setting : Other (malls, military units, universities, camps, centers, etc)** | | | | | | | | | | | | |
| KSA | Al-Asmary, S. M. et al (2008) ^(38)^ | 1238  *(only males)* | 19-56 |  | 24.4 |  |  |  |  |  | 71.5 |  |
|  | Koura, M. R. et al (2012) ^(39)^ | 370  *(only females)* | 19.9.± 1.4 |  | 40.0 |  |  |  |  |  |  |  |
|  | Amin, T. T.et al (2014) ^(40)^ | 691 | 24-63 |  | 72.5 |  |  |  |  |  |  |  |
| Lebanon | Matar, D.et al (2015) ^(41)^ | 1697 | >=21 |  | 53.0 |  | 48.9 | 93.0 |  | 27.0 |  | 54.2 |
| UAE | Shah. S.M. et al (2015) [^‖^](https://en.wikipedia.org/wiki/Vertical_Bar)^(42)^ | 1375  *(only males)* | >=18 |  | 23.6 |  |  | 48.5 |  |  |  | 8.3 |
|  | Yusufali, A. M. et al (2015) ^(43)^ | 4128 | >=18 |  | 56.0 |  |  |  |  |  | 48.0 |  |

| **Country** | **First Author (year)** | **Sample size** | **Age**  *(age group or*  *mean age± SD)* |  | **Prevalence of awareness (%)**  **among hypertensives** |  | **Prevalence of treatment (%)** | |  | **Prevalence of control (%)** | | |
| --- | --- | --- | --- | --- | --- | --- | --- | --- | --- | --- | --- | --- |
|  |  |  |  |  |  |  | **Among hypertensives** | **Among aware hypertensives** |  | **Among hypertensives** | **Among aware hypertensives** | **Among treated hypertensives** |
| **PANEL B: CLINICAL POPULATION (HYPERTENSIVES)** | | | | | | | | | | | | |
| Algeria | Ragot, S. et al (2016) ^§(44)^ | 505 | 62.7.± 10.2 |  |  |  |  |  |  |  |  | 57.0 |
| Bahrain | Al Khaja, K. A. J.et al (2004) ^(45)^ | 225 | 60-80 |  |  |  |  | 96.4 |  |  |  | 15.2 |
| Egypt | Youssef, R. M. et al (2005) ^(46)^ | 316 | 35-83 |  |  |  |  |  |  |  | 53.2 |  |
|  | Ragot, S.et al (2016) ^§(44)^ | 464 | 55.2.± 10.2 |  |  |  |  |  |  |  |  | 40.9 |
| Jordan | Khasawneh, N.F.et al (2005) ^(47)^ | 207 | >=51 |  |  |  |  |  |  |  | 59.4 |  |
|  | Khader, A.et al (2014) ^¶(48)^ | 18881 | >=18 |  |  |  |  |  |  |  | 83.0 |  |
| KSA | Siddiqui, S.et al (2001) ^(49)^ | 3747 | >=18 |  |  |  |  | 91.7 |  |  | 31.0 |  |
|  | Al-Tuwirji, A. A.et al (2006) ^(50)^ | 255 | >=18 |  |  |  |  |  |  |  | 40.4 |  |
| Lebanon | Yusef, J.I et al (2000) ^¶(51)^ | 2202 | NA |  |  |  |  |  |  |  | 75.7 |  |
|  | Tohme, R. A. et al (2005) ^**(52)^ | 2010 | >=30 |  |  |  |  | 89.4 |  |  |  |  |
|  | Mallat, S. G. et al (2014) ^**(53)^ | 988 | >=18 |  |  |  |  |  |  |  | 67.52 |  |
| Morocco | Habbal, R.et al (2010) ^(54)^ | 457 | >=18 |  |  |  |  |  |  |  | 18.6 |  |
| Oman | El-Badawy, A. M.et al (2005) ^(55)^ | 100 | >=20 |  |  |  |  |  |  |  | 27.0 |  |
| Sudan | Elzubier, A. G. et al (2000) ^(56)^ | 198 | 53.03.± 11.2 |  |  |  |  |  |  |  | 61.6 |  |
|  | Babiker, F. A. et al (2013) ^(57)^ | 200 | >=20 |  |  |  |  | 82.0 |  |  | 64.0 |  |
| Tunisia | Ben Abdelaziz, A. et al (2007) ^(58)^ | 456 | 41-92 |  |  |  |  |  |  |  | 11.2 |  |
|  | Masmoudi, J.et al (2010) ^(59)^ | 100 | 61.85.± 11.95 |  |  |  |  |  |  |  | 35.0 |  |

DBP: diastolic blood pressure; F: Females; M: Males; NA: Not available; SBP: systolic blood pressure

*Awareness, treatment and control were defined as reported by the authors in the original publications. In general, awareness was defined as self-report of any prior diagnosis of hypertension; treatment of hypertension was defined as use of a medication for management of high blood pressure; and control of hypertension was defined as having an average SBP <140mmHg and an average DBP <90mmHg. Some authors used different denominators to assess prevalence of undiagnosed/unawareness of hypertension which prevented the reviewers from calculating the prevalence of awareness among hypertensives. In Sudan, undiagnosed hypertension was estimated at 38.2% (60, 61) and 49.4% (62) in adult subjects with no previous history of HTN. In Egypt, undiagnosed hypertension was estimated at 11% among those who did not give history of hypertension (AbdelAziz et al, 2015) (63). In KSA undiagnosed systolic hypertension (BP>140mm Hg) and diastolic hypertension (BP >90 mmHg) was detected in 11.6% and 11% of adult subjects with no previous history of HTN (64). In KSA, Al Zahrani et al (2011) (65), reported the rate of awareness (34%) among patients with high blood pressure readings (i.e. >=140/90 mmHg) and not among hypertension patients. Similarly, in KSA, Al-Turki, Y. A. et al (2015) (66) reported the percentage of participants diagnosed as hypertensives and taking anti-hypertensive medications among all the study population and not among hypertensives;

**Nationally representative studies;

§ Multi-country studies: Yusuafli et al (2017) includes data from Palestine, KSA and UAE and is part of the PURE study; Nejjari et al (2013) includes pooled data from Algeria (n=11,950), Morocco (n=10,714) and Tunisia (n=5,881); Ragot et al (2016) includes data from five countries: Algeria, Pakistan, Ukraine, Egypt and Venezuela (Only control rates reported in Algeria and Egypt were retrieved by the reviewer). In Alsheik et al (2014) hypertension was recorded in 43% of outpatients (n=4378) from 14 African and Middle Eastern countries (including Cameroon, Ghana, Tunisia, Algeria, Senegal, KSA, Kenya, South Africa, Nigeria, UAE, Egypt, Lebanon, Jordan, and Kuwait); pooled data are only presented and thus are not included in the table. Results showed that 33% of all patients were treated with antihypertensive drugs, 76.3% of hypertensives were treated, 47% of treated hypertensives had controlled blood pressure, and 59.6% of hypertensives had controlled blood pressure (67). Chow et al (2013) is also based on the PURE study and reported the pooled prevalence rates of awareness (52.5%), treatment (50.8%) and control (17.1%) among hypertensives for Middle Eastern countries (including Iran, Turkey and UAE) (68)

† Weighted prevalence rates were generated in the original publication.

†† Weighted prevalence of unawareness (75.5%) and uncontrolled blood pressure (65.8%) were reported in the original publication. The treatment coverage of self-reported hypertensive patients during the 12 months preceding the survey was 82.7%.

‡ Crude prevalence of awareness, treatment and control were retrieved from the Supplementary Table 1 (Yusufali et al, 2017)

∫The study sample consists of elderly populations from Algeria and Europe. The reviewers only retrieved the values for Algeria.

[‖](https://en.wikipedia.org/wiki/Vertical_Bar)Sample consisted of South Asian immigrant males (Indian, Pakistani and Bangladeshi) in Al Ain, UAE.

¶Samples consisted of hypertensives Palestinian refugees at UNRWA Primary health care centers in Jordan (Khader et al, 2014) and Lebanon (Yusef et al, 2000)

**References:**

1. Temmar M, Labat C, Benkhedda S, Charifi M, Thomas F, Bouafia MT, et al. Prevalence and determinants of hypertension in the Algerian Sahara. Journal of hypertension. 2007;25(11):2218-26.

2. Hamida F, Atif ML, Temmar M, Chibane A, Bezzaoucha A, Bouafia MT. [Prevalence of hypertension in El-Menia oasis, Algeria, and metabolic characteristics in population]. Annales de Cardiologie et d Angeiologie. 2013;62(3):172-8.

3. Al-Mahroos F, Al-Roomi K, McKeigue PM. Relation of high blood pressure to glucose intolerance, plasma lipids and educational status in an Arabian Gulf population. International Journal of Epidemiology. 2000;29(1):71-6.

4. Mohamed MR, Shafek M, El Damaty S, Seoudi S. Hypertension control indicators among rural population in Egypt. Journal of the Egyptian Public Health Association. 2000;75(5-6):391-401.

5. Jaddou HY, Bateiha AM, Ajlouni KM. Prevalence, awareness and management of hypertension in a recently urbanised community, eastern Jordan. Journal of human hypertension. 2000;14(8):497-501.

6. Jaddou HY, Bateiha AM, Al-Khateeb MS, Ajlouni KM. Epidemiology and management of hypertension among Bedouins in Northern Jordan. Saudi Medical Journal. 2003;24(5):472-6.

7. Shakhatreh FM, Suleiman AA, Mohammed FI, Alwan AA. Hypertension among females in a highly disadvantaged community in Jordan. Health Care for Women International. 2008;29(1):39-53.

8. Jaddou H, Batieha A, Khader YS, Kanaan A, El-Khateeb M, Ajlouni K. Hypertension prevalence, awareness, treatment and control, and associated factors: results from a national survey, Jordan. International journal of hypertension. 2011;2011.

9. Kheirallah KA, Liswi M, Alazab R, Bataineh Z, Alzyoud S, Alsulaiman J, et al. Hypertension Prevalence, Awareness and Control Levels among Ghawarna: An African-Descendant Ethnic Minority in the Jordan Valley. Ethnicity & Disease. 2015;25(3):321-8.

10. Al-Nozha MM, Abdullah M, Arafah MR, Khalil MZ, Khan NB, Al-Mazrou YY, et al. Hypertension in Saudi Arabia. Saudi Medical Journal. 2007;28(1):77-84.

11. Saeed AA, Al-Hamdan NA, Bahnassy AA, Abdalla AM, Abbas MA, Abuzaid LZ. Prevalence, Awareness, Treatment, and Control of Hypertension among Saudi Adult Population: A National Survey. International journal of hypertension. 2011;2011:174135.

12. El Bcheraoui C, Memish ZA, Tuffaha M, Daoud F, Robinson M, Jaber S, et al. Hypertension and its associated risk factors in the kingdom of saudi arabia, 2013: a national survey. International journal of hypertension. 2014;2014:564679.

13. Mirza AA, Elmorsy SA. Diagnosis and Control of Hypertension as Indicators of the Level of Awareness Among Relatives of Medical Students in Saudi Arabia. High Blood Pressure & Cardiovascular Prevention. 2016;23(2):123-32.

14. Yusufali AM, Khatib R, Islam S, Alhabib KF, Bahonar A, Swidan HM, et al. Prevalence, awareness, treatment and control of hypertension in four Middle East countries. Journal of hypertension. 2017;35(7):1457-64.

15. Tazi MA, Abir-Khalil S, Lahmouz F, Arrach ML, Chaouki N. Risk factors for hypertension among the adult Moroccan population. Eastern Mediterranean Health Journal. 2009;15(4):827-41.

16. Al Riyami AA, Afifi M. Clustering of cardiovascular risk factors among Omani adults. Eastern Mediterranean health journal = La revue de sante de la Mediterranee orientale = al-Majallah al-sihhiyah li-sharq al-mutawassit. 2003;9(5-6):893-903.

17. Abd El-Aty MA, Meky FA, Morsi MM, Al-Lawati JA, El Sayed MK. Hypertension in the adult Omani population: predictors for unawareness and uncontrolled hypertension. J Egypt Public Health Assoc. 2015;90(3):125-32.

18. Maziak W, Rastam S, Mzayek F, Ward KD, Eissenberg T, Keil U. Cardiovascular health among adults in Syria: a model from developing countries. Annals of Epidemiology. 2007;17(9):713-20.

19. Ben Romdhane H, Skhiri H, Bougatef S, Ennigrou S, Gharbi D, Chahed MK, et al. [Hypertension prevalence, awareness, treatment and control: results from a community based survey]. Tunisie Medicale. 2005;83 Suppl 5:41-6.

20. Hammami S, Mehri S, Hajem S, Koubaa N, Frih MA, Kammoun S, et al. Awareness, treatment and control of hypertension among the elderly living in their home in Tunisia. BMC Cardiovascular Disorders. 2011;11:65.

21. Ben Romdhane H, Ben Ali S, Skhiri H, Traissac P, Bougatef S, Maire B, et al. Hypertension among Tunisian adults: results of the TAHINA project. Hypertension Research - Clinical & Experimental. 2012;35(3):341-7.

22. Gunaid AA, Assabri AM. Prevalence of type 2 diabetes and other cardiovascular risk factors in a semirural area in Yemen. Eastern Mediterranean Health Journal. 2008;14(1):42-56.

23. Modesti PA, Bamoshmoosh M, Rapi S, Massetti L, Al-Hidabi D, Al Goshae H. Epidemiology of hypertension in Yemen: effects of urbanization and geographical area. Hypertension Research - Clinical & Experimental. 2013;36(8):711-7.

24. Temmar M, Watfa G, Joly L, Kearney-Schwartz A, Youcef M, Bensalah S, et al. Elderly Algerian women lose their sex-advantage in terms of arterial stiffness and cardiovascular profile. Journal of hypertension. 2013;31(11):2244-50.

25. Bachir Cherif A, Bouamra A, Taleb A, Bouraghda A, Rabia S, Imouloudene N, et al. [The characteristics of arterial hypertension in postmenopausal women in the area of Blida (Algeria)]. Annales de Cardiologie et d Angeiologie. 2016;65(3):146-51.

26. Whitford DL, Al-Anjawi HA, Al-Baharna MM. Impact of clinical inertia on cardiovascular risk factors in patients with diabetes. Primary care diabetes. 2014;8(2):133-8.

27. Al-Homrany MA, Khan MY, Al-Khaldi YM, Al-Gelban KS, Al-Amri HS. Hypertension care at primary health care centers: a report from Abha, Saudi Arabia. Saudi Journal of Kidney Diseases & Transplantation. 2008;19(6):990-6.

28. Kalantan KA, Mohamed AG, Al-Taweel AA, Abdul Ghani HM. Hypertension among attendants of primary health care centers in Al-Qassim region, Saudi Arabia. Saudi Medical Journal. 2001;22(11):960-3.

29. Awad AI, Alsaleh FM. 10-year risk estimation for type 2 diabetes mellitus and coronary heart disease in Kuwait: a cross-sectional population-based study. PLoS ONE [Electronic Resource]. 2015;10(1):e0116742.

30. Arevian M, Adra M, Kubeissi L. Risk factors for coronary artery disease (CAD) in Lebanese-Armenian women. Health Care for Women International. 2004;25(10):933-49.

31. Barakat MN, Youssef RM. Prevalence of dysglycemia and other cardiovascular risk factors among the rural population of Oman. Saudi Medical Journal. 2008;29(12):1824-6.

32. Khdour MR, Hallak HO, Shaeen M, Jarab AS, Al-Shahed QN. Prevalence, awareness, treatment and control of hypertension in the Palestinian population. Journal of human hypertension. 2013;27(10):623-8.

33. Abdelsatir S, Al-Sofi A, Elamin S, Abu-Aisha H. The potential role of nursing students in the implementation of community-based hypertension screening programs in Sudan. Arab Journal of Nephrology and Transplantation. 2013;6(1):51-4.

34. Laouani Kechrid C, Hmouda H, Ben Naceur MH, Ghannem H, Toumi S, Ajmi F. [High blood presure for people aged more than 60 years in the distrct of Sousse]. Tunisie Medicale. 2004;82(11):1001-5.

35. Abdulle AM, Nagelkerke NJ, Abouchacra S, Pathan JY, Adem A, Obineche EN. Under- treatment and under diagnosis of hypertension: a serious problem in the United Arab Emirates. BMC Cardiovascular Disorders. 2006;6:24.

36. Noman O, Al-Kaddoomi SA, Del Ben M, Angelico F. Impact of urbanization on the prevalence and pattern of arterial hypertension on the island of Socotra. Annals of Saudi Medicine. 2008;28(2):143-4.

37. Nejjari C, Arharbi M, Chentir MT, Boujnah R, Kemmou O, Megdiche H, et al. Epidemiological Trial of Hypertension in North Africa (ETHNA): an international multicentre study in Algeria, Morocco and Tunisia. Journal of hypertension. 2013;31(1):49-62.

38. Al-Asmary SM, Al-Shehri AA, Farahat FM, Abdel-Fattah MM, Al-Shahrani MM, Al-Omari FK, et al. Community-based screening for pre-hypertension among military active duty personnel. Saudi Medical Journal. 2008;29(12):1779-84.

39. Koura MR, Al-Dabal BK, Rasheed P, Al-Sowielem LS, Makki SM. Prehypertension among young adult females in Dammam, Saudi Arabia. Eastern Mediterranean Health Journal. 2012;18(7):728-34.

40. Amin TT, Al Sultan AI, Mostafa OA, Darwish AA, Al-Naboli MR. Profile of non-communicable disease risk factors among employees at a Saudi university. Asian Pacific Journal of Cancer Prevention: Apjcp. 2014;15(18):7897-907.

41. Matar D, Frangieh AH, Abouassi S, Bteich F, Saleh A, Salame E, et al. Prevalence, awareness, treatment, and control of hypertension in Lebanon. Journal of clinical hypertension (Greenwich, Conn). 2015;17(5):381-8.

42. Shah SM, Loney T, Sheek-Hussein M, El Sadig M, Al Dhaheri S, El Barazi I, et al. Hypertension prevalence, awareness, treatment, and control, in male South Asian immigrants in the United Arab Emirates: a cross-sectional study. BMC Cardiovascular Disorders. 2015;15:30.

43. Yusufali A, Bazargani N, Muhammed K, Gabroun A, AlMazrooei A, Agrawal A, et al. Opportunistic Screening for CVD Risk Factors: The Dubai Shopping for Cardiovascular Risk Study (DISCOVERY). Global heart. 2015;10(4):265-72.

44. Ragot S, Beneteau M, Guillou-Bonnici F, Herpin D. Prevalence and management of hypertensive patients in clinical practice: Cross-sectional registry in five countries outside the European Union. Blood Pressure. 2016;25(2):104-16.

45. Al Khaja KA, Sequeira RP, Damanhori AH. Pharmacotherapy and blood pressure control in elderly hypertensives in a primary care setting in Bahrain. Aging-Clinical & Experimental Research. 2004;16(4):319-25.

46. Youssef RM, Moubarak, II, Kamel MI. Factors affecting the quality of life of hypertensive patients. Eastern Mediterranean Health Journal. 2005;11(1-2):109-18.

47. Khasawneh NF, Al-Safi S, Albsoul-Younes A, Borqan ON. Clustering of coronary artery disease risk factors in Jordanian hypertensive patients. Saudi Medical Journal. 2005;26(2):215-9.

48. Khader A, Farajallah L, Shahin Y, Hababeh M, Abu-Zayed I, Zachariah R, et al. Hypertension and treatment outcomes in Palestine refugees in United Nations Relief and Works Agency primary health care clinics in Jordan. Tropical Medicine & International Health. 2014;19(10):1276-83.

49. Siddiqui S, Ogbeide DO, Karim A, Al-Khalifa I. Hypertension control in a community health centre at Riyadh, Saudi Arabia. Saudi Medical Journal. 2001;22(1):49-52.

50. Al-Tuwijri AA, Al-Rukban MO. Hypertension control and co-morbidities in primary health care centers in Riyadh. Annals of Saudi Medicine. 2006;26(4):266-71.

51. Yusef JI. Management of diabetes mellitus and hypertension at UNRWA primary health care facilities in Lebanon. Eastern Mediterranean Health Journal. 2000;6(2-3):378-90.

52. Tohme RA, Jurjus AR, Estephan A. The prevalence of hypertension and its association with other cardiovascular disease risk factors in a representative sample of the Lebanese population. Journal of human hypertension. 2005;19(11):861-8.

53. Mallat SG, Samra SA, Younes F, Sawaya MT. Identifying predictors of blood pressure control in the Lebanese population - a national, multicentric survey -- I-PREDICT. BMC public health. 2014;14:1142.

54. Habbal R, Sekhri AR, Volpe M, i-Search I. Prevalence of microalbuminuria in hypertensive patients and its associated cardiovascular risk in clinical cardiology: Moroccan results of the global i-SEARCH survey - a sub-analysis of a survey with 21,050 patients in 26 countries worldwide. Cardiovascular Journal of Africa. 2010;21(4):200-5.

55. El-Badawy AM, Al-Kharusi HM, Al-Ghanemy SA. Health habits and risk factors among Omanis with hypertension. Saudi Medical Journal. 2005;26(4):623-9.

56. Elzubier AG, Husain AA, Suleiman IA, Hamid ZA. Drug compliance among hypertensive patients in Kassala, eastern Sudan. Eastern Mediterranean health journal = La revue de sante de la Mediterranee orientale = al-Majallah al-sihhiyah li-sharq al-mutawassit. 2000;6(1):100-5.

57. Babiker FA, Elkhalifa LA, Moukhyer ME. Awareness of hypertension and factors associated with uncontrolled hypertension in Sudanese adults. Cardiovascular Journal of Africa. 2013;24(6):208-12.

58. Ben Abdelaziz A, Gaha K, Elrafei W, Mandhouj O, Ben Othman A, Ghannem H. [Predictors of increased cardiovascular risk in hypertensive patients in frontline facilities in Sousse, Tunisia]. Eastern Mediterranean Health Journal. 2007;13(2):319-25.

59. Masmoudi J, Imene T, Ketata W, Mnif L, Maalej S, Kammoun S, et al. [Role of the psychosocial factors in blood pression balance; cross-sectional study including 100 ambulatory hypertensive patients]. Tunisie Medicale. 2010;88(4):223-9.

60. Bushara SO, Noor SK, Elmadhoun WM, Sulaiman AA, Ahmed MH. Undiagnosed hypertension in a rural community in Sudan and association with some features of the metabolic syndrome: how serious is the situation? Renal Failure. 2015;37(6):1022-6.

61. Noor SK, Bushara SO, Sulaiman AA, Elmadhoun WM. A preliminary survey of un-diagnosed hypertension among Nubians and Coptics in Atbara and Eldamer Cities, Sudan: does ethnicity affect prevalence? Arab Journal of Nephrology and Transplantation. 2013;6(3):193-5.

62. Noor SK, Elsugud NA, Bushara SO, Elmadhoun WM, Ahmed MH. High prevalence of hypertension among an ethnic group in Sudan: implications for prevention. Renal Failure. 2016;38(3):352-6.

63. Abd Elaziz KM, Dewedar SA, Sabbour S, El Gafaary MM, Marzouk DM, Aboul Fotouh A, et al. Screening for hypertension among adults: community outreach in Cairo, Egypt. J Public Health (Oxf). 2015;37(4):701-6.

64. Alam AA, Mitwalli AH, Al-Wakeel JS, Chaudhary AR, Zebaid MA. Plasma fibrinogen and its correlates in adult Saudi population. Saudi Medical Journal. 2004;25(11):1593-602.

65. Al-Zahrani MS. Prehypertension and undiagnosed hypertension in a sample of dental school female patients. International Journal of Dental Hygiene. 2011;9(1):74-8.

66. Al Turki YA. Blood pressure status during consultation: a primary care study. High Blood Pressure & Cardiovascular Prevention. 2015;22(1):79-82.

67. Alsheikh-Ali AA, Omar MI, Raal FJ, Rashed W, Hamoui O, Kane A, et al. Cardiovascular risk factor burden in Africa and the Middle East: the Africa Middle East Cardiovascular Epidemiological (ACE) study. PLoS ONE [Electronic Resource]. 2014;9(8):e102830.

68. Chow CK, Teo KK, Rangarajan S, Islam S, Gupta R, Avezum A, et al. Prevalence, awareness, treatment, and control of hypertension in rural and urban communities in high-, middle-, and low-income countries. Jama. 2013;310(9):959-68.
