# Supplementary material for: The cascade of care in managing hypertension in the Arab world: a systematic assessment of the evidence on awareness, treatment and control
Source: BMC Public Health. 2020 Jun 3;20:835. doi: 10.1186/s12889-020-08678-6 (PMC7268748; doi:10.1186/s12889-020-08678-6)
Supplement: Supplementary file 3 — Additional file 3. Supplementary Table 2 (Table S2). Studies of hypertension awareness, treatment and control in the Arab countries, 2000–2017 (percent among respondents by stage and relative percent losses between stages). Studies of hypertension awareness, treatment and control in the Arab countries, 2000–2017 (percent among respondents by stage and relative percent losses between stages). [file 12889_2020_8678_MOESM3_ESM.docx]

**Additional file 3**

**Supplementary Table 2 (Table S2): Studies of hypertension awareness, treatment and control in the Arab countries, 2000-2017 (percents among respondents by stage and relative-percents losses between stages)**

| **Study** | **Sample size** | **Age ^a^** | **Prevalence^b^ (%)** | **Among Hypertensives ^c^** | | | | | | | |
| --- | --- | --- | --- | --- | --- | --- | --- | --- | --- | --- | --- |
|  |  |  |  | **Loss 1 (%)** | | **Aware (%)** | **Loss 2 (%)** | **Treated(%)** | | **Loss 3 (%)** | **Controlled (%)** |
| **Household** | | | | | | | | | | | |
| **Algeria** |  |  |  |  |  | |  |  |  | |  |
| Hamidaa,F. et al (2013) ^(46)^ | 722 | >=40 | 50.0 | *- 57.5* | 42.5 | | *-4* | 40.8 | *-79.6* | | 8.3 |
| **Bahrain** |  |  |  |  |  | |  |  |  | |  |
| Al-Mahroos, F. et al (2000) ^e^ ^(43)^ | 2090 | 40-69 | 30 | *-38.0* | 62 | | *-10.2* | 55.7 | *-75.9* | | 13.4 |
| **Jordan** |  |  |  |  |  | |  |  |  | |  |
| Jaddou, H.Y. et al (2011)^f (40)^ | 4117 | >=25 | 32.3 | *- 43.9* | 56.1 | | *-36.7* | 35.5^g^ | *-60.4* | | 14.07^g^ |
| Kheirallah, K. A et al (2015) ^h (41)^ | 517^g^ | >=25 | 44.3 | *-63.8* | 36.2 | | *-8.3* | 33.2^g^ | *-69.9* | | 10.0 ^g^ |
| **KSA** |  |  |  |  |  | |  |  |  | |  |
| Saeed, A.A. et al (2011) ^f (45)^ | 4758 | 15-64 | 25.5 | *- 55.3* | 44.7 | | *-28.2* | 32.1^g^ | *-62.9* | | 11.9 ^g^ |
| El-Bcheraoui C. et al (2014) ^f (44)^ | 10735 | >=15 | 15.2 | *- 57.8* | 42.2 ^i^ | | *-12.8* | 36.8 ^i^ | *-54.9* | | 16.6 ^i^ |
| Yusufali, A. M. et al (2017) ^j (25)^ | 1545 | 35-70 | 30.0 | *-39.0* | 61.0^g^ | | *-3.3* | 59.0^gf^ | *-49.2* | | 30.0 ^g^ |
| **Morocco** |  |  |  |  |  | |  |  |  | |  |
| Tazi, M.A. et al. (2009) ^f (28)^ | 1802 | >=20 | 39.6 | *- 78.1* | 21.9 | | *-59.8* | 8.8 | *-87.5* | | 1.1 |
| **Palestine** |  |  |  |  |  | |  |  |  | |  |
| Yusufali, A. M. et al (2017) ^j (25)^ | 1545 | 35-70 | 37.0 | *-40.0* | 60.0 | | *-3.3* | 58.0 ^g^ | *-60.3* | | 23.0 ^g^ |
| **Tunisia** |  |  |  |  |  | |  |  |  | |  |
| Ben Romdhane, H.et al. (2005) ^(31)^ | 1837 | 40-69 | 44.3 | *- 58.1* | 41.9 | | *-26.0* | 31.0 ^g^ | *-86.8* | | 4.1^g^ |
| Hammami, S. et al. (2011)^(36)^ | 598 | >=65 | 52.0 | *- 19.0* | 81.0 | | *-3.2* | 78.4 | *-68.9* | | 24.4 ^g^ |
| Ben Romdhane, H. et al. (2012) ^f (47)^ | 8007 | 35-74 | 30.6 | *- 61.2* | 38.8 | | *-15.2* | 32.9 ^g^ | *-76.0* | | 7.9 |
| **UAE** |  |  |  |  |  | |  |  |  | |  |
| Yusufali, A. M. et al (2017) ^j (25)^ | 1545 | 35-70 | 52.0 | *-48.0* | 52.0 | | *-3.9* | 50.0 ^g^ | *-74.0* | | 13.0^g^ |
| **Yemen** |  |  |  |  |  | |  |  |  | |  |
| Modesti, P. A. et al. (2013) ^f (20)^ | 10242 | 15-69 | 12.8 | *- 55.5* | 44.5 | | *-9.2* | 40.4 | *-76.0* | | 9.7^g^ |
| **Health care facilities** | | | | | | | | | | | |
| **Kuwait** |  |  |  |  |  | |  |  |  | |  |
| Awad, A. I& Alsaleh, F. (2015) ^f (33)^ | 1,610 | 20-79 | 20.0 | *-41.9* | 58.1 ^f^ | | *0* | 58.1^g^ | *-33.2* | | 38.8 ^g^ |
| **Palestine (West Bank)** |  |  |  |  |  | |  |  |  | |  |
| Khdour, M. R. et al. (2013)^(48)^ | 2,077 | 25-92 | 27.6 | *-49* | 51.0 | | *-21.2* | 40.2 | *-75.9* | | 9.7 |
| **Sudan** |  |  |  |  |  | |  |  |  | |  |
| Abdelsatir, S. et al. (2013)^(38)^ | 389 | 41 ± 15 | 39.6 | *-27.9* | 72.1 | | *-23.7* | 55.0^g^ | *-36.2* | | 35.1 |
| **Tunisia** |  |  |  |  |  | |  |  |  | |  |
| Laouani Kechrid,C.et al. (2004) ^(37)^ | 600 | >=60 | 69.3 | *-31.5* | 68.5 | | *-3.1* | 66.4 | *-76.2* | | 15.8 |
| **Yemen** |  |  |  |  |  | |  |  |  | |  |
| Noman, O. et al. (2008)^(42)^ | 994 | NA | 22.3^i^ | *-63.8* | 36.2 ^i^ | | *-15.5* | 30.6^i^ | *-11.8* | | 27.0 ^i^ |
| **Multicountry study** | |  |  |  |  | |  |  |  | |  |
| Nejjari, C. et al. (2013) ^(39)^ | 28,500^j^ | >=18 | 45.4 | *-54.8* | 71.0 | | *-8.7* | 64.8 ^g^ | *-53.2* | | 30.3^g^ |
| **Other (malls, plazas, centers)^l^** | | | | | | | | | | | |
| **Lebanon** | | | |  |  | |  |  |  | |  |
| Matar, D. et al. (2015) ^(35)^ | 1,697 | >=21 | 36.9 | *-47.0* | 53.0 | | *-7.7* | *48.9* | *-44.8* | | 27.0 |
|  |  |  |  |  |  | |  |  |  | |  |
|  |  |  |  |  |  | |  |  |  | |  |

NA: Not available

a. Number of BP measurements was not mentioned in three studies (Al-Mahrous et al, 2000; Jaddou et al, 2011; Noman et al, 2008). For the other studies, BP were recorded as the average of a) 2 BP measurements (AbdelSatir et al, 2013; Awad & Alsaleh, 2015; Ben Romdhane et al, 2005; Ben Romdhane et al, 2012; Hammami et al, 2011; Khdour et al, 2013; Kheirallah et al, 2015; Nejjari et al, 2013; Shah et al, 2015; Yusufali et al, 2017), b) the last 2 BP measurements out of 3 (Hamidaa et al, 2013; Tazi et al, 2009), c) 3 BP measurements (El-Bcheraoui et al, 2014; Louani Kechrid et al, 2004; Saeed et al, 2011), d) 2 or 3BP (Matar et al, 2015), e) 4 BP out of 6 (Modesti et al, 2013).

b. Age is reported in years either as age group or as mean age ± standard deviation; one study did not report the sample population’s age (Noman et al, 2008).

c. Prevalence of hypertension was identified when participants reported (1) being on current anti-hypertensive drugs and/or (2) having blood pressure measures of SBP >=160 mmHg and/or DBP >=95 mmHg for Al-Mahroos et al (2000); SBP >=140 mmHg and/or DBP >=80 mmHg for Kheirallah et al (2015); and SBP >=140 mmHg and/or DBP >=90mmHg for all the remaining studies. Awareness, treatment and control were defined as the authors reported them in the original publications

d. Proportion of patients lost when moving from one stage to another (relative difference), calculated as follows:

Loss 1 (%) = (% aware - all hypertensives (i.e. 100%)) / all hypertensives; Loss 2 (%) = (% treated - % aware)/% aware; and Loss 3 (%) = (% controlled - % treated)/ % treated.

e. Invitations were sent to individuals at households inviting them to participate in a screening survey at health centers

f. Nationally representative studies.

g. Estimates, based on our calculations.

h. Sample consisted of Ghawarna (an African-Descendant Ethnic Minority) living in Jordan.

i. Awareness, treatment and contol rates are weighted in the original publications (El-Bcheraoui et al, 2014; Noman et al, 2008).

j. Crude prevalence of awareness, treatment and control retrieved from the Supplementary Table 1 (Yusufali et al, 2017)

k. Total sample size was 28,500: Algeria (n=11,905); Morocco (n=10,714) & Tunisia (n=5,881).

l. A study conducted by Shah et al (2015) at a government VISA screening center reported simultaneously rates of awareness, treatment and control of hypertension. However, as the sample consists of South Asian male immigrants solely and not general populations, we did not report the results in this table.
